# Gut Microbiota and Autism Spectrum Disorders: Neurodevelopmental, Behavioral, and Gastrointestinal Interactions

**DOI:** 10.3390/nu17172781

**Published:** 2025-08-27

**Authors:** Zuzanna Lewandowska-Pietruszka, Magdalena Figlerowicz, Katarzyna Mazur-Melewska

**Affiliations:** 1 Poznan University of Medical Sciences, Department of Infectious Diseases and Child Neurology, 60-572 Poznan, Poland; lewandowska.pietruszka@gmail.com (Z.L.-P.);; 2 Poznan University of Medical Sciences, Doctoral School, 60-812 Poznan, Poland

**Keywords:** autism spectrum disorder, gut microbiota, gut-brain axis, functional gastrointestinal disorder, diet, behavior

## Abstract

Background: Autism spectrum disorder (ASD) is a complex neurodevelopmental condition characterized by social communication deficits, repetitive behaviors, and frequent gastrointestinal comorbidities. Emerging research suggests gut microbiota alterations contribute to ASD symptoms and gastrointestinal dysfunction, but detailed microbial profiles and clinical correlations remain underexplored. Methods: This study analyzed gut microbiota in 45 children aged 2–18 years diagnosed with ASD. Stool samples underwent 16S rRNA gene sequencing. Clinical assessments included ASD diagnostic subtype, adaptive functioning using the Vineland Adaptive Behavior Scale, gastrointestinal symptoms as per the Rome IV criteria, dietary patterns, and demographic variables. Statistical analyses correlated microbiota profiles with clinical features. Results: Gut microbiota composition was significantly influenced by delivery mode, age, sex, and diet. Vaginally delivered children had higher beneficial SCFA-producing bacteria, whereas Cesarean section was linked to increased pathogenic *Clostridiales*. High-calorie and protein-rich diets correlated with shifts toward pro-inflammatory taxa. Microbial diversity and specific genera correlated with adaptive behavior domains (communication, socialization, motor skills) and severity of gastrointestinal symptoms. Both pro-inflammatory and anti-inflammatory bacteria variably impacted neurodevelopmental outcomes. Conclusions: Gut microbiota composition in children with ASD is shaped by multifactorial influences and connected to neurobehavioral and gastrointestinal phenotypes. The findings of this study support the potential of microbiota-targeted interventions to ameliorate ASD-associated symptoms and improve quality of life.

## 1. Introduction

Autism spectrum disorder (ASD) is a multi-etiological disorder impacting the neurological development of individuals. The World Health Organization estimates that approximately 1 in 100 children globally exhibits symptoms of autism; however, prevalence data from developing countries may be underreported [1,2]. ASD is defined by its core symptoms, including differences in social and communicative function, stereotypical patterns of behavior, and selective interests [3]. Alongside the core symptoms, patients often present with comorbidities. These include neuropsychological conditions such as intellectual disability, anxiety, affective disorders, sleep disturbances, and epilepsy. Somatic conditions are also common, particularly physical disabilities and gastrointestinal symptoms [4]. The latter are observed in up to 70% of patients and can lead to a significant reduction in quality of life [5].

A growing body of evidence supports the role of abnormalities in the intestinal microbiome in core and associated symptoms [6,7]. The results remain equivocal; however, there are several patterns observed in multiple studies on microbiota composition in patients with ASD. Children with ASD present with lower abundance of Bacteroidetes, alongside higher abundance of *Firmicutes* and *Pseudomonadota* [8]. The bacteria from genera *Prevotella*, *Roseburia*, *Ruminococcus*, *Megasphaera*, and *Catenibacterium* were hypothesized to be potential biomarkers for ASD [8,9,10]. A lower *Bacteroidetes* to *Firmicutes* ratio and higher abundance of *Desulfovibrio*, *Bacteroides*, and *Clostridium* were observed in patients with more severe core symptoms. A lower *Bacteroidetes* to *Firmicutes* ratio, typically observed in patients with ASD, seems to correlate with gastrointestinal and behavioral symptoms [8,11,12]. Moreover, diet composition, including elimination diets or food selectivity (FS), has a visible impact on microbiota composition in patients with ASD [8,13,14,15].

Furthermore, microbiota composition can influence hormonal responses, while hormonal stress response leads to differences in microbiota composition [16,17,18]. Abnormalities in the release of one of the main stress hormones, cortisol, were previously observed in children with ASD. Patients presented with an abnormally low basal level of cortisol and an atypical hormonal response to stressors. A common phenomenon was a flattened diurnal cortisol release slope [19].

We proposed that children with ASD may exhibit unique gut microbiota profiles, linked to both gastrointestinal and behavioral characteristics, and that these microbial patterns are associated with dietary habits. The results of this study could also serve as an initial step toward developing an interventional microbiota method of reducing the gastrointestinal or affective symptoms reported by patients as being difficult to bear, potentially leading to an increase in quality of life.

The specific objectives of this study were to: (1) define the intestinal microbiota composition in children with ASD through 16S rRNA gene sequencing; (2) examine associations between microbiome composition, dietary patterns, food preferences, and gastrointestinal symptoms; (3) assess correlations between gut microbial profiles and adaptive functioning; and (4) identify microbial markers with potential predictive value for functional outcomes in ASD.

## 2. Materials and Methods

### 2.1. Participants

Participants were enrolled by the research team from the Department of Infectious Diseases and Child Neurology at Poznan University of Medical Sciences between 2020 and 2023. Eligibility criteria were: (1) a confirmed clinical ASD diagnosis, i.e., established by a multidisciplinary team composed of psychologists and psychiatrists, in line with ICD-10 guidelines, and (2) age ranging from 2 to 18 years. Initially, 234 children’s guardians expressed interest in being included in the research group.

Exclusion criteria comprised: (1) inability or refusal to complete all required questionnaires and/or provide biological samples within the study timeline; (2) epilepsy diagnosis, as confirmed by a pediatric neurologist; (3) confirmed organic gastrointestinal disorders, as assessed by a pediatric gastroenterologist; (4) use within the past three months of medications likely to alter gut microbiota, including antibiotics, probiotics, or acid-suppressing agents; (5) history of fecal microbiota transplantation; and (6) currently following an elimination diet due to conditions such as celiac disease, lactose intolerance, or allergies, as validated by a specialist.

Exclusions were applied as follows: 59 for incomplete participation or unwillingness to comply with study requirements; 18 owing to epilepsy; 15 due to gastrointestinal disorders; 68 due to recent pharmacological treatments; and 29 because of elimination diets. After these exclusions, the final cohort comprised 45 patients, including 7 female and 38 male participants. Informed consent was obtained from legal guardians for the collection of clinical data and biological samples.

### 2.2. The Evaluation of Medical Background

Diagnosis as per ICD-10 (F84.0 versus other subtypes of ASD), age, sex, mode of delivery, and coexisting conditions were stated by guardians and their children on a personal questionnaire.

The patients’ functioning was measured using the Vineland Adaptive Behavior Scale, Third Edition (VABS), with scores examined in relation to the primary domains of socialization, communication, daily living skills, and motor abilities. Additionally, subdomains such as receptive, expressive, and written communication, daily living skills, socialization, and gross and fine motor skills were evaluated, enabling the identification of groups based on their functional levels.

A questionnaire was administered to guardians to evaluate symptoms of functional gastrointestinal disorders, in accordance with the Rome IV Criteria.

Parents were asked to keep a detailed dietary log over a period of seven days, noting the child’s food intake, which included five main meals as well as any snacks and drinks consumed. When relevant, brand names were specified. A standardized paper form was provided for each participant for manual completion. In addition to recording the types of food, caregivers estimated portion sizes and documented the timing of each eating occasion [20,21]. Using this information, the researchers calculated calorie and fluid consumption, along with the nutritional breakdown of fats (saturated and unsaturated), sugars (simple and complex), and proteins. Based on their dietary profiles, participants were categorized into six groups according to their intake patterns: low-calorie diet (LCD), defined by calorie consumption not surpassing standard age- and sex-specific recommendations; high-calorie diet (HCD), with intake above typical levels; protein-based diet (PBD), where protein made up more than 20–30% of daily energy; and carbohydrate-based diet (ChBD).

### 2.3. Collecting and Storing Stool Samples

The lead researcher collected stool specimens individually from each child in an interior environment. Just before collecting, the child’s vital measurements were recorded, and information regarding recent exposure to sick individuals was gathered. Sterile stool collection containers (F.L. Medical s.r.l., Torreglia, Italy) were used for sampling. All specimens were transferred to a specialized laboratory freezer (ARCTIKO Ltd., Salisbury, UK) and stored at −80 °C within one hour after collection. To protect patient privacy and reduce bias, each sample was anonymized by the investigator through the use of a unique, coded identifier.

### 2.4. The Analysis of Microbiome Composition

Genomic DNA extraction was performed by skilled laboratory staff using the Auto-Pure Mini system (Allsheng, Hangzhou, China) along with MagnifiQ reagents (A&A Biotechnology, Gdańsk, Poland). DNA libraries were created via PCR amplification specifically targeting the V3–V4 regions of the 16S rRNA gene. Microbial community profiling was then carried out using Next-Generation Sequencing (NGS) of bacterial 16S rRNA genes on the Illumina MiSeq platform (Illumina, San Diego, CA, USA). All DNA samples that met the quality standards required for 16S rRNA gene sequencing were included in the study; no samples were excluded due to insufficient yield or degradation. The same bioinformatics workflow was applied uniformly to all samples.

Sequence quality control was conducted through the DADA2 method implemented within QIIME 2. During denoising, forward and reverse reads were truncated at 260 base pairs, with the first 10 bases trimmed from forward reads and the first 20 bases from reverse reads. A maximum of two expected errors per read was allowed, and reads were trimmed at the initial base where the quality score dropped below 2. Chimeric sequences were identified and removed using a consensus-based approach. Only sequence variants with a total frequency of at least 10 across the dataset were preserved for further analysis.

To eliminate non-bacterial sequences, taxonomic filtering was applied: features assigned to mitochondria, chloroplasts, Archaea, or Eukaryota were excluded. Taxonomic classification used QIIME 2′s classify-consensus-blast against the SILVA 138-99 reference database.

All experimental work was carried out at the certified SANPROBI Research and Development Centre in Szczecin, Poland, under the guidance of expert scientists associated with Pomeranian Medical University, Szczecin.

### 2.5. Statistics and Biostatistics

Data analyses were performed by a skilled biostatistician using “R” version 4.3.2 (released on 30 October 2023, ucrt). Relationships between different microbiota compositions and influencing factors were examined through Spearman’s and Winsorized Pearson’s correlation methods. Multivariate relationships within the microbiota data were explored using Pearson’s correlation. The choice between Pearson’s and Spearman’s correlation tests was guided by the assessment of bivariate normality with the Shapiro–Wilk test. A *p*-value threshold of ≤0.05 was applied to determine statistical significance.

### 2.6. Ethical Approval

All procedures involving human participants were performed in full compliance with the ethical principles outlined by the relevant institutional and/or national research ethics committees and were consistent with the 1964 Declaration of Helsinki, including its subsequent revisions and comparable international guidelines [22]. The study protocol underwent formal review and received approval from the Bioethics Committee of Poznań University of Medical Sciences (approval no. KB 1138/19; 5 December 2019). Prior to study participation, written informed consent was obtained from all individuals and/or their legal representatives, ensuring voluntary enrollment and adherence to ethical requirements.

## 3. Results

### 3.1. Gut Microbiota Sequencing

The analysis of the gut microbiota using stool DNA samples exposed the existence of microbial communities that encompassed 11 phyla, 16 classes, 42 orders, 83 families, 223 genera, and 363 species. Consequently, associations between the microbiota diversity and various clinical, dietary, and physiological characteristics of the study population were examined in subsequent analyses. Researchers found several statistically significant links between the types of microbes in a child’s gut and their health and nutrition. Robust correlations (r > 0.5 or r < −0.5) were regarded as potentially biologically relevant. The composition of the microbiota varied substantially among participants; nonetheless, certain patterns were evident in most children. The predominant bacteria belonged to the *Clostridium* class, particularly the genera *Bacilli* and *Subdoligranulum*, and *Actinobacteria*, mainly the *Bifidobacterium* genus. Although there is no established consensus on the normal *Firmicutes* to *Bacteroidetes* ratio, it was notably low across all participants compared to existing data (mean 0.04 ± 0.14). Further analyses explored the relationships between microbiota diversity at various taxonomic levels and a range of clinical, dietary, and physiological factors in the study group, including the presence of functional gastrointestinal disorders (FGID), dietary habits and food selectivity (FS), and participants’ performance across multiple functional domains.

### 3.2. The Structure of the Group

The cohort consisted of 45 children with ASD, with a marked male predominance (n = 38, 84%) compared to females (n = 7, 16%), which aligns with the known higher prevalence of ASD in males. The participants were stratified by age into three categories: preschoolers (2–6 years; n = 12), school children (6–14 years; n = 28), and adolescents (15–18 years; n = 5). Regarding perinatal factors, vaginal delivery was reported in 69% of the cases (n = 31), while Cesarean section occurred in 31% (n = 14) of cases. A detailed analysis of the structure of the group is shown in Table 1.

**Figure 1:**
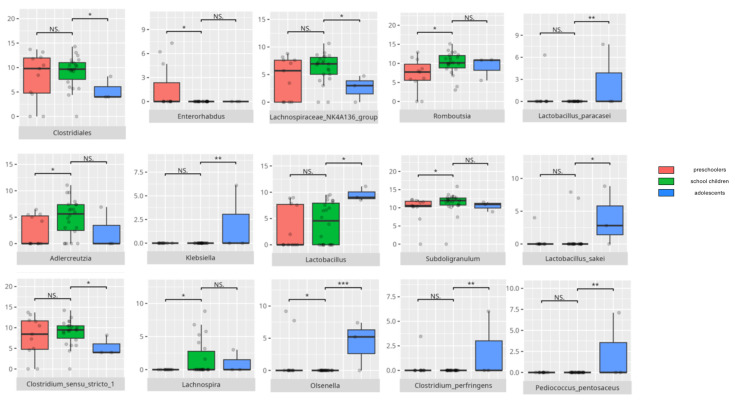
Statistically significant differences among age groups (* *p* ≤ 0.05, ** *p* ≤ 0.01, *** *p* ≤ 0.001, NS: not significant).

**Figure 2:**
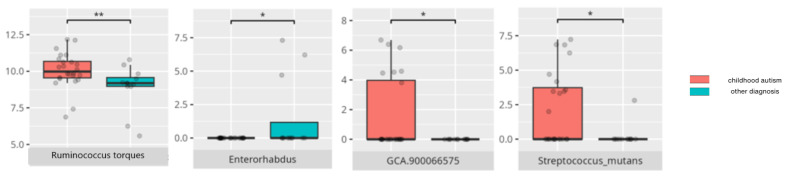
Statistically significant differences between the two diagnosis groups (* *p* ≤ 0.05, ** *p* ≤ 0.01).

**Figure 3:**
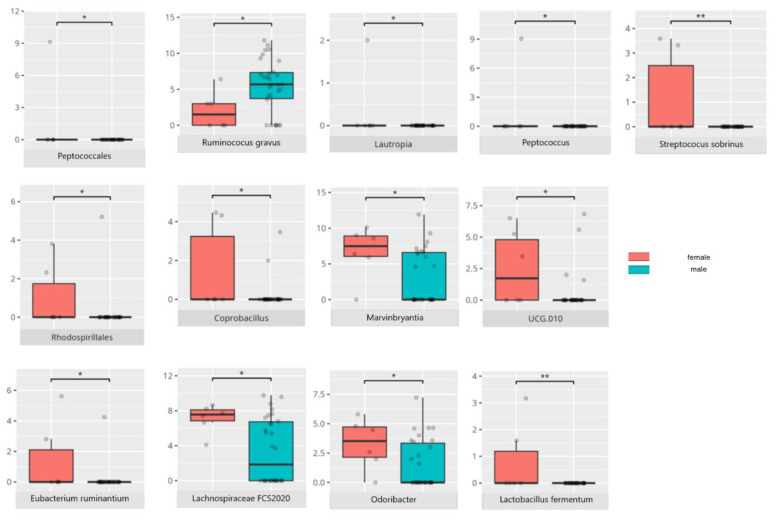
Statistically meaningful differences between the two sex groups (* *p* ≤ 0.05, ** *p* ≤ 0.01).

**Figure 4:**
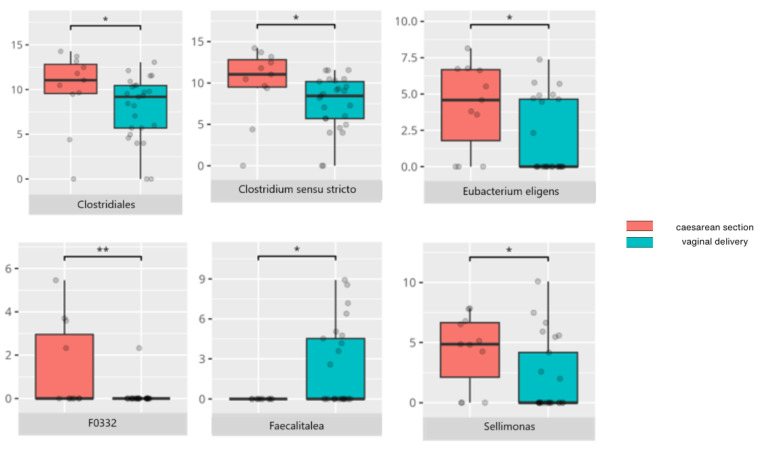
Statistically significant differences between the two modes of delivery groups (* *p* ≤ 0.05, ** *p* ≤ 0.01).

There were notable disparities in the gut microbiota composition between the different participant subgroups. The statistically significant differences are detailed in Table 2 and illustrated in Figure 1, Figure 2, Figure 3 and Figure 4.

*Micrococcales* abundance was negatively correlated with age (r = −0.47), while age exhibited a negative correlation with *Rothia* abundance (r = −0.47). Table 3 displays all the correlations.

### 3.3. Diet and Food Selectivity

The analysis of the data on diet composition is shown in Table 1.

ChBD was declared by the parents of 50% (n = 6) of preschoolers, 25% (n = 7) of school children, and only one adolescent. Most of them (n = 12, 86%) were boys and did not present with symptoms of FGID (n = 8, 57%).

HCD was consumed by all of the preschoolers (n = 12), 32% (n = 9) of the school children, and only one adolescent. The majority of them (n = 19, 86%) were boys and did not present with symptoms of FGID (n = 14, 63%).

Most of the children with FS were boys (n = 13, 81%) of preschool age (n = 10, 63%). Twenty-five percent (n = 4) of them also presented with FGID symptoms. Sixty-nine percent (n = 11) of the children with FS had HCD, while the numbers following a protein-based and ChBD were balanced.

**Figure 5:**
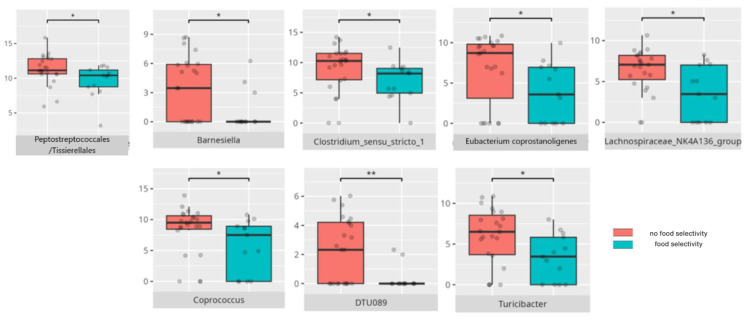
Statistically meaningful differences between the two food selectivity groups (* *p* ≤ 0.05, ** *p* ≤ 0.01).

**Figure 6:**
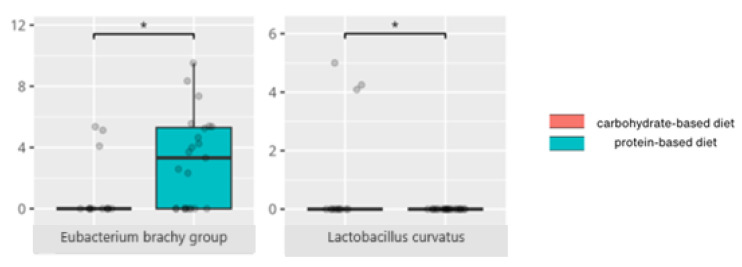
Statistically significant differences between two diet subtypes—carbohydrate-based diet vs. protein-based diet (* *p* ≤ 0.05).

**Figure 7:**
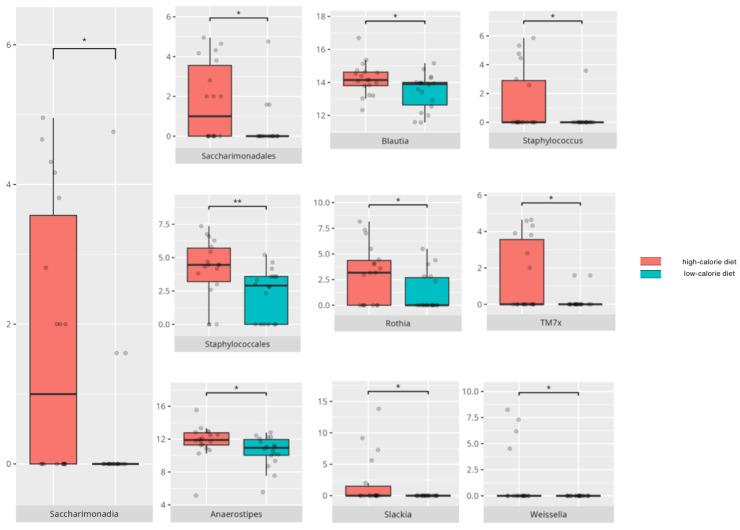
Statistically significant differences between two diet subtypes—high-calorie diet vs. low-calorie diet (* *p* ≤ 0.05, ** *p* ≤ 0.01).

Gut microbiota compositions differed due to diet differences. The statistically important differences are detailed in Table 2 and illustrated in Figure 5, Figure 6 and Figure 7.

The ratio between saturated fat and total fat correlated negatively with *Peptostreptococcales/Tissierellales* abundance (r = −0.46). The mean consumption of complex carbohydrates correlated positively with the *Clostridiales* (r = 0.5) and *Peptostreptococcales/Tissierellales* (r = 0.47) levels.

At the genus level, the ratio between saturated fat and total fat consumption positively correlated with *Leuconostoc* (r = 0.49) abundance, while it correlated negatively with *Eubacterium coprostanoligenes* (r = −0.46), *Eubacterium hallii* (r = −0.59), and *Dorea* (r = −0.6) abundance. On the other hand, the unsaturated fat to total fat intake ratio (r = 0.55) and mean fat consumption to weight ratio (r = 0.47) correlated positively with *Dielma* abundance. The simple carbohydrates to total carbohydrates intake ratio positively correlated with *Epulopiscium* (r = 0.53) and *Weissella* (r = 0.51) abundance and negatively with *Flavonifractor* abundance (r = −0.5). Complex carbohydrates intake correlated positively with *Romboutsia* level (r = 0.52). The ratios between the energy obtained from carbohydrates to total energy consumed and between the energy gained from carbohydrates to total carbohydrates intake both correlated positively with *Epulopiscium* (r = 0.5) and *Klebsiella* (r = 0.66) abundance, with the latter correlating positively also with the *Weissella* level (r = 0.51). *Dielma* abundance was positively correlated with mean protein intake to weight ratio (r = 0.52).

The saturated fat to total fat intake ratio positively correlated with *Enterobacter cloacae* (r = 0.68), *Leuconostoc citreum* (r = 0.53), *Leuconostoc lactis* (r = 0.56), *Staphylococcus epidermidis* (r = 0.46), and *Streptococcus gallolyticus* (r = 0.66) abundance. The ratio between kilocalories from carbohydrates and mean total carbohydrates intake positively correlated with *Clostridum perfingens* abundance (r = 0.62).

Table 3 contains a list of all correlations.

### 3.4. Functional Gastrointestinal Disorders

**Figure 8:**
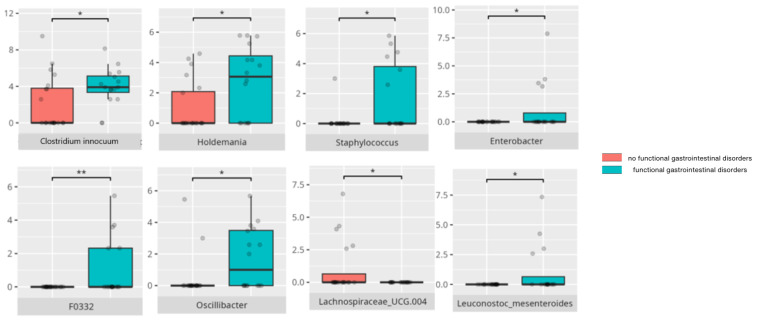
Statistically relevant differences between the two functional gastrointestinal disorder groups (* *p* ≤ 0.05, ** *p* ≤ 0.01).

**Figure 9:**
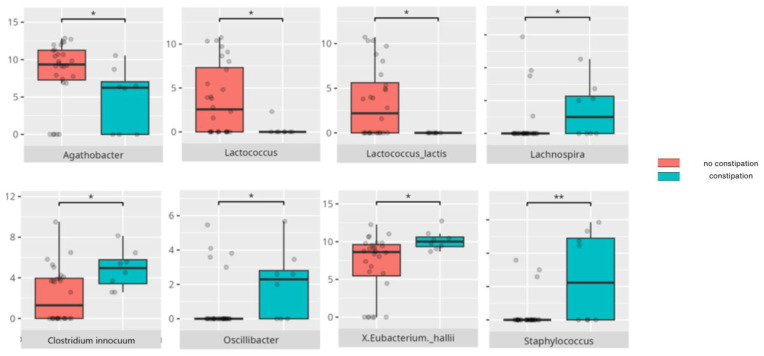
Statistically meaningful differences between the two groups (* *p* ≤ 0.05, ** *p* ≤ 0.01).

**Figure 10:**
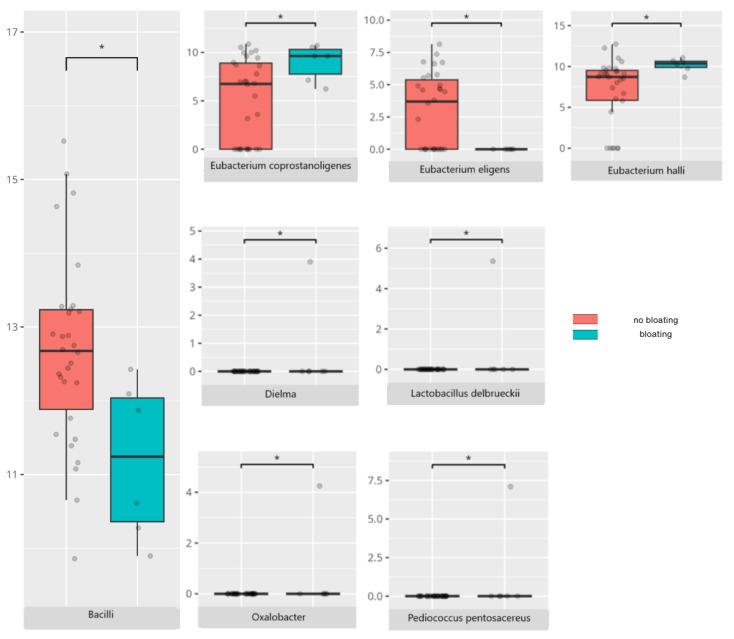
Statistically significant differences between the two groups (* *p* ≤ 0.05).

**Figure 11:**
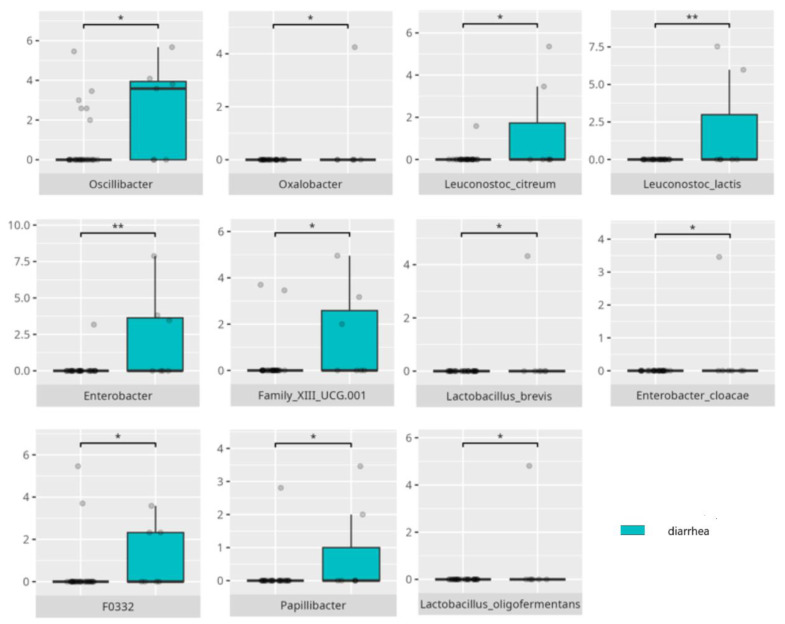
Statistically relevant differences between the two groups (* *p* ≤ 0.05, ** *p* ≤ 0.01).

The data on the prevalence of FGID in the research group are shown in Table 1. The group presenting with any FGID consisted of four preschoolers (33%), 11 school children (39%), and three adolescents (60%), mostly boys (n = 16, 89%). Significant differences were observed in the constitution of the gut microbiota depending on the occurrence of FGID. The statistically important differences are detailed in Table 2 and illustrated in Figure 8, Figure 9, Figure 10 and Figure 11.

### 3.5. Vineland Adaptive Behavioral Scale Results

All patients were assessed for their functioning across four domains, i.e., communication, daily living skills, socialization, and motor skills, and specific subdomains were further explored. We compared three groups of children—those with low, adequate, or high scores in each domain and subdomain—against microbiota abundance at the different taxa levels, emphasizing statistically significant distinctions. Additionally, we examined the correlations between gut microbiota constitution and different factors, including age, dietary preferences, weight, and the V-scores from the VABS assessment.

#### 3.5.1. Domain: Communication

**Figure 12:**
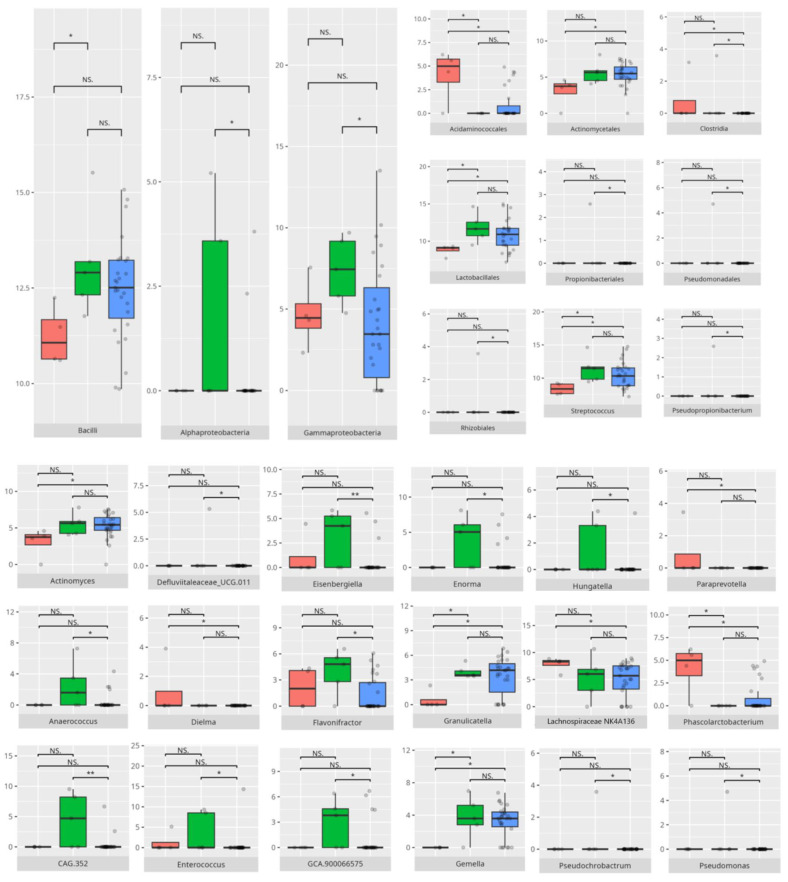

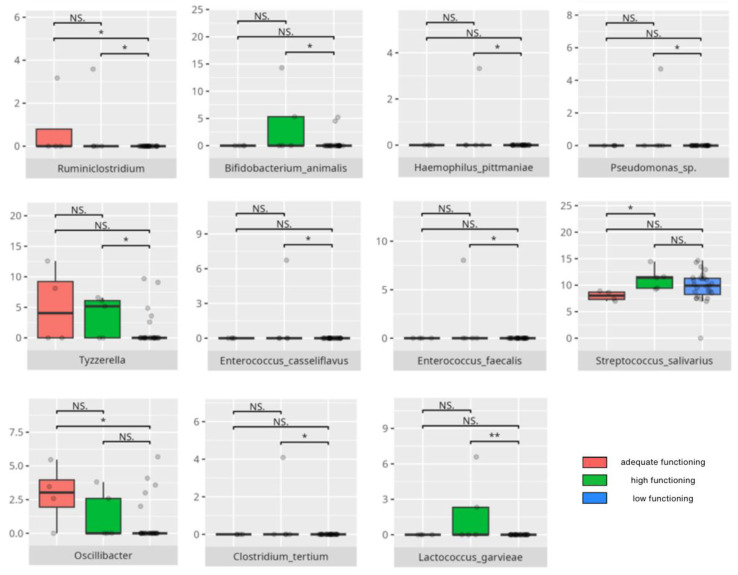
Statistically significant differences between the two groups in the communication domain (* *p* ≤ 0.05, ** *p* ≤ 0.01, NS: not significant).

Thirty-one children received a low or moderately low result in the total communication domain. Four participants received an adequate result, while 10 received a high or moderately high result. Statistically important differences are detailed in Table 2 and illustrated in Figure 12.

##### Subdomain: Receptive Communication

**Figure 13:**
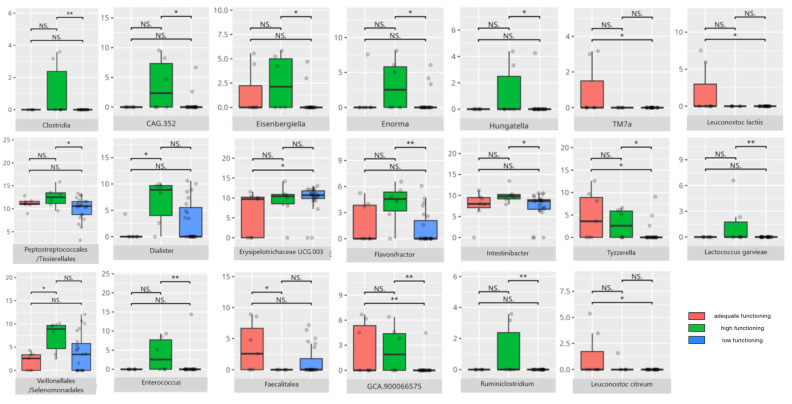
Statistically meaningful differences between the two groups in the receptive communication subdomain (* *p* ≤ 0.05, ** *p* ≤ 0.01, NS: not significant).

In the receptive communication subdomain, 10 participants demonstrated high or moderately high scores, whereas 27 exhibited low or moderately low performance. Among the higher-scoring group, seven were from the middle group and three were older boys. FGID were present in three individuals, and FS was reported in another three. Most followed PBD (n = 9) and LCD (n = 8). The lower-scoring subgroup included 11 preschoolers, 15 school children, and one adolescent. FGID were identified in 10 participants, and FS was observed in 11. The majority followed PBD (n = 17) and HCD (n = 15) dietary patterns. Statistically important differences are detailed in Table 2 and illustrated in Figure 13. V-score of receptive communication positively correlated with *Flavonifractor* abundance (r = 0.49). Table 3 displays all the correlations.

##### Subdomain: Expressive Communication

In the expressive communication subdomain, 10 children (seven boys and one girl) achieved high or moderately high scores, whereas 24 children obtained low or moderately low scores. Among those with higher scores, eight belonged to the school children group and two were older adolescents. Two of these participants presented with FGID, and three with FS. The majority adhered to PBD (n = 9) or LCD (n = 8).

Children with lower scores were predominantly boys (n = 20). This group comprised eight preschoolers, 14 school-aged children, and two adolescents. Nine participants presented with one or more FGIDs, and 11 with FS. Most of them followed PBD (n = 14), and half also adhered to LCD.

**Figure 14:**
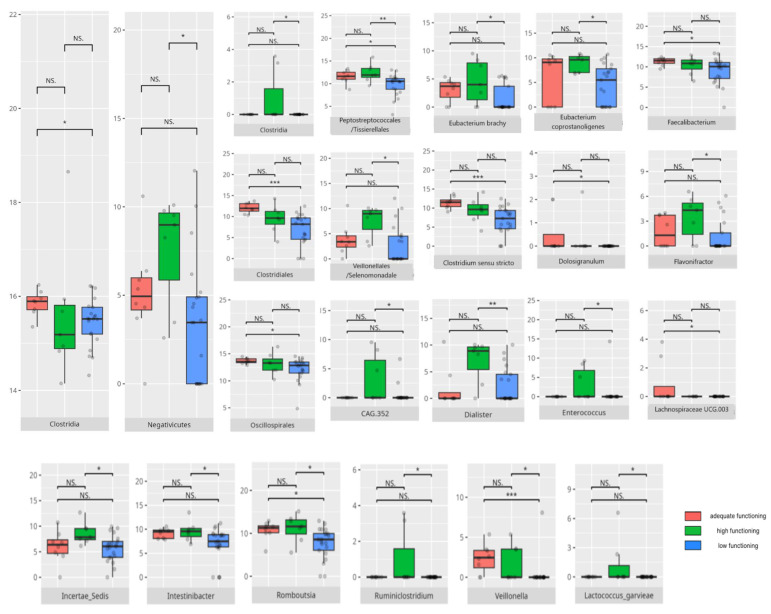
Statistically relevant differences between the two groups in the expressive communication subdomain (* *p* ≤ 0.05, ** *p* ≤ 0.01, *** *p* ≤ 0.001, NS: not significant).

Statistically important differences are detailed in Table 2 and illustrated in Figure 14.

##### Subdomain: Writing Skills

Within the writing skills subdomain, 5 participants attained scores in the high or moderately high range, whereas 32 demonstrated weaker performance. The higher-achieving group consisted exclusively of boys from the middle age category; two of these children presented with FGID and one also with FS. Three followed PBD, while every child in this subgroup adhered to LCD.

Among those with lower outcomes, all 16 were school boys. Nine presented with FGID and 8 with FS. Dietary assessments revealed that 15 children followed PBD, and 12 also reported adherence to LCD.

**Figure 15:**
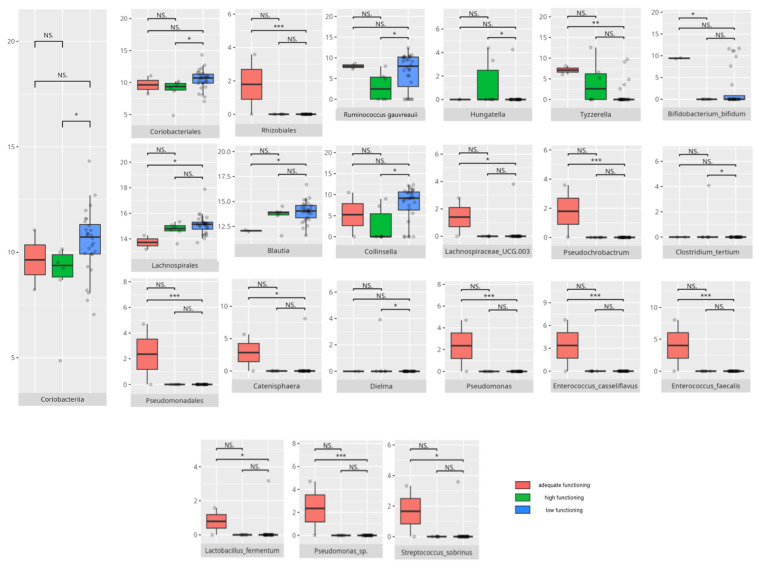
Statistically significant differences between the two groups in the writing skills subdomain (* *p* ≤ 0.05, ** *p* ≤ 0.01, *** *p* ≤ 0.001, NS: not significant).

The statistically significant differences are detailed in Table 2 and illustrated in Figure 15. V-score for writing skills negatively correlated with the abundance of *Anaerostipes* (r = −0.51). Table 3 contains a list of all correlations.

#### 3.5.2. Domain: Daily Living Skills

**Figure 16:**
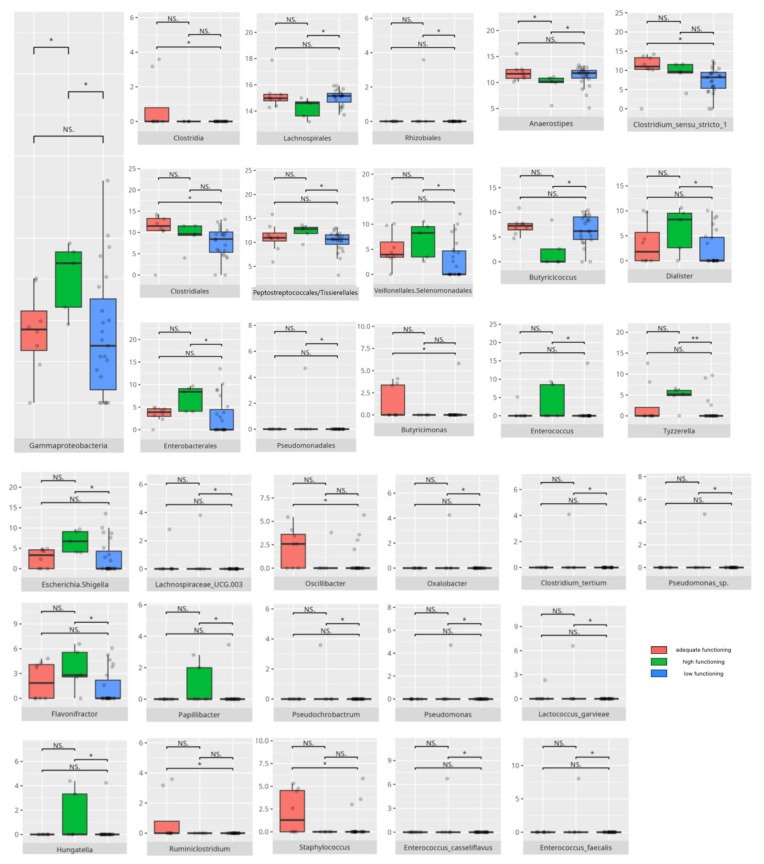
Statistically relevant differences between the two groups in the daily living skills domain (* *p* ≤ 0.05, ** *p* ≤ 0.01, NS: not significant).

In the total daily living skills domain, 27 children obtained low or moderately low scores. Ten participants achieved results within the adequate range, while 8 demonstrated high or moderately high performance. Statistically significant differences are presented in Table 2 and graphically depicted in Figure 16.

##### Subdomain: Personal Skills

**Figure 17:**
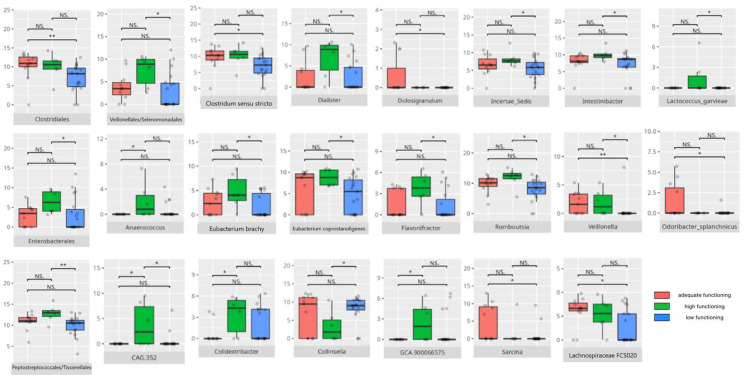
Statistically significant differences between the two groups in the personal skills subdomain (* *p* ≤ 0.05, ** *p* ≤ 0.01, NS: not significant).

Eight participants achieved high or moderately high scores in the personal skills subdomain, while 27 scored low or moderately low. All of the high scorers were boys, including six school-aged children and two adolescents. Among them, five had been diagnosed with childhood autism. Two had FGID, and three had FS. Most of these participants consumed PBD (n = 6) and LCD (n = 7). The lower-scoring group consisted mainly of boys (n = 20), with eight preschoolers, 14 school-aged children, and two adolescents. Six in this group had diagnoses other than childhood autism. Nine had FGID, and 11 had FS. The majority followed PBD (n = 14), and 11 also consumed LCD. Statistically significant differences are presented in Table 2 and illustrated in Figure 17. The V-score in the personal skills subdomain showed a positive correlation with *Romboutsia* abundance (r = 0.5). Table 3 displays all the correlations.

##### Subdomain: Domestic Skills

**Figure 18:**
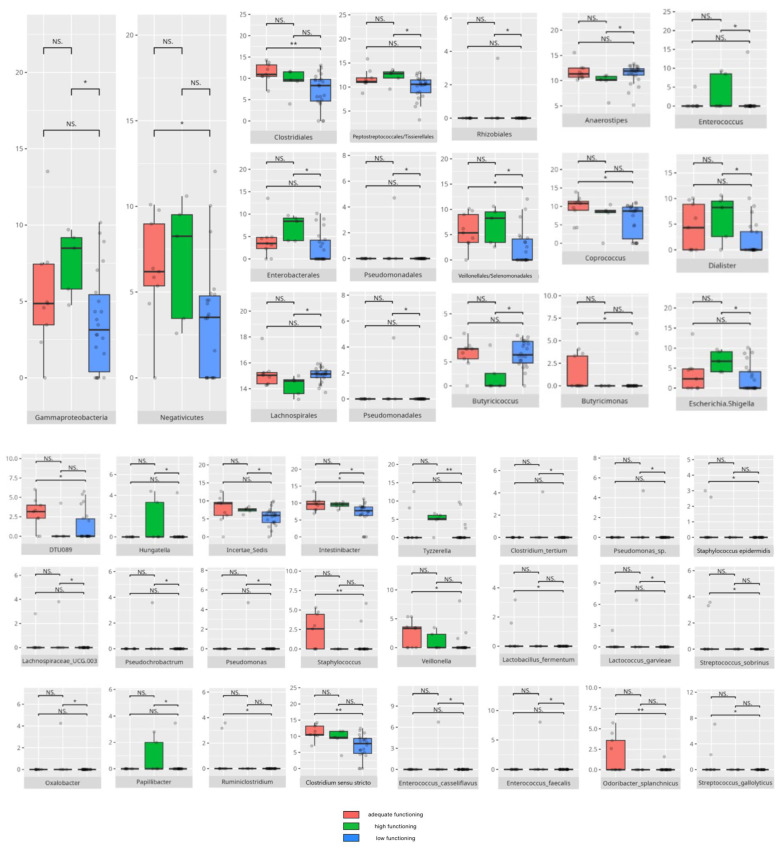
Statistically meaningful differences between the two groups in the domestic skills subdomain (* *p* ≤ 0.05, ** *p* ≤ 0.01, NS: not significant).

Seven children (all boys) achieved high or moderately high scores in the domestic skills subdomain, while 27 children (24 boys and three girls) scored low or moderately low. Among the high scorers, five were from the intermediate age group and two were older children. Two of these children had one or more FGIDs, and two had FS. Most followed PBD (n = 5) and LCD (n = 7). The lower-scoring group included seven preschoolers, 17 school-aged children, and three adolescents. Eight of them had diagnoses other than childhood autism. Eleven had any FGID, and 13 had FS. The majority of this group consumed PBD (n = 18) and HCD (n = 14). Statistically significant differences are presented in Table 2 and depicted in Figure 18. The V-score in the domestic skills subdomain showed a positive correlation with *Intestinibacter* abundance (r = 0.5).

##### Subdomain: Community Skills

**Figure 19:**
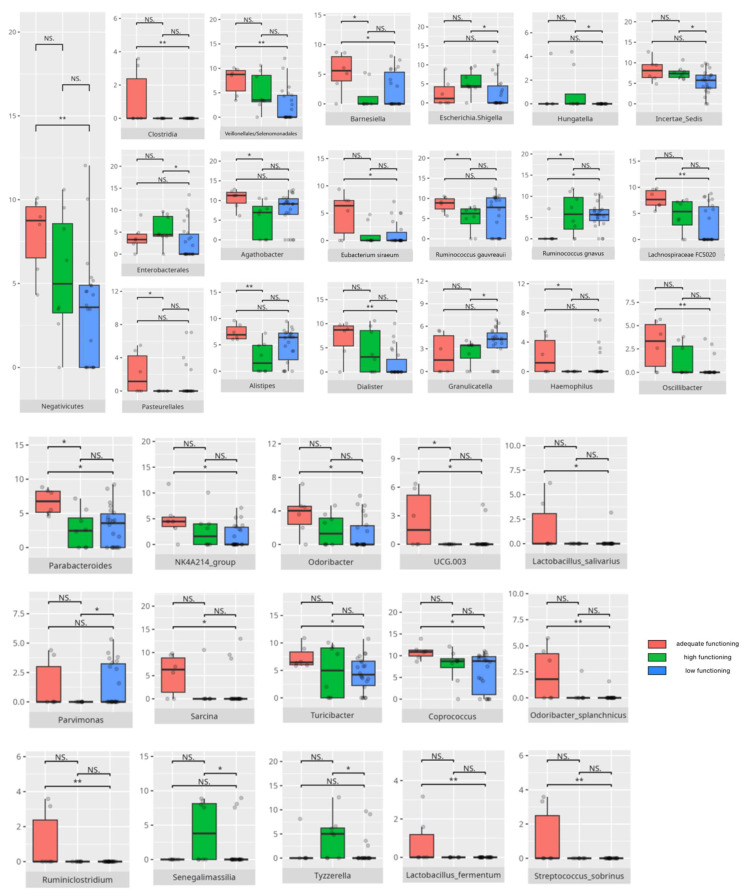
Statistically relevant differences between the two groups in the community skills subdomain (* *p* ≤ 0.05, ** *p* ≤ 0.01, NS: not significant).

Eleven children scored high or moderately high in the community skills subdomain, while 26 scored low or moderately low. Among the higher scorers, two were preschoolers, seven were school-aged children, and two were adolescents. Nine of these children were boys, and five had been diagnosed with childhood autism. Six presented with one or more FGIDs, and two also had FS. Nine followed PBD, and nine adhered to LCD. Of the lower-scoring group, ten were preschoolers, 14 were school-aged children, and two were adolescents, with 22 boys in total. Nineteen children in this group were diagnosed with childhood autism. Nine had any FGID, and 12 had FS. Fifteen participants consumed PBD, while 16 followed HCD. The statistically significant differences are detailed in Table 2 and illustrated in Figure 19.

#### 3.5.3. Domain: Socialization

**Figure 20:**
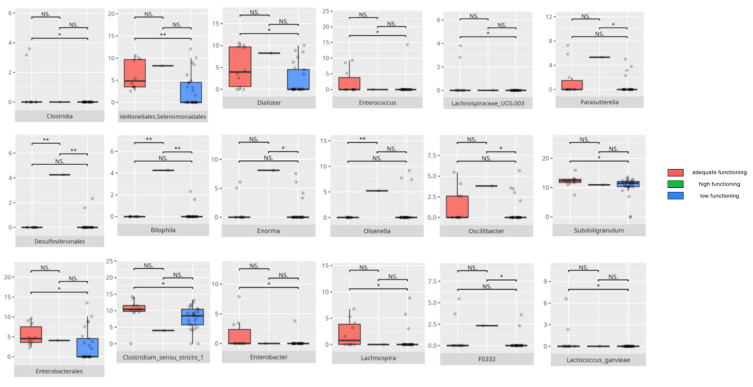
Statistically significant differences between the two groups in the socialization domain (* *p* ≤ 0.05, ** *p* ≤ 0.01, NS: not significant).

Thirty children received a low or moderately low result in total in the socialization domain. Eleven participants received an adequate result, while four received a high or moderately high result. The statistically important differences are detailed in Table 2 and illustrated in Figure 20.

##### Subdomain: Interpersonal Skills

Six participants scored high or moderately high in the interpersonal skills subdomain, while 28 scored low or moderately low. All of the high scorers were boys, including one preschooler, three school-aged children, and two adolescents. Three of them were diagnosed with childhood autism. Three presented with one or more FGIDs, and two had FS. Half of these participants followed PBD (n = 3), and most consumed LCD (n = 5). Among the lower-scoring group, most were boys (n = 23). Eleven were preschoolers, 14 were school-aged children, and three were adolescents. Ten had diagnoses other than childhood autism. Twelve presented with one or more FGIDs, and twelve had FS. The majority consumed PBD (n = 19) and HCD (n = 16).

**Figure 21:**
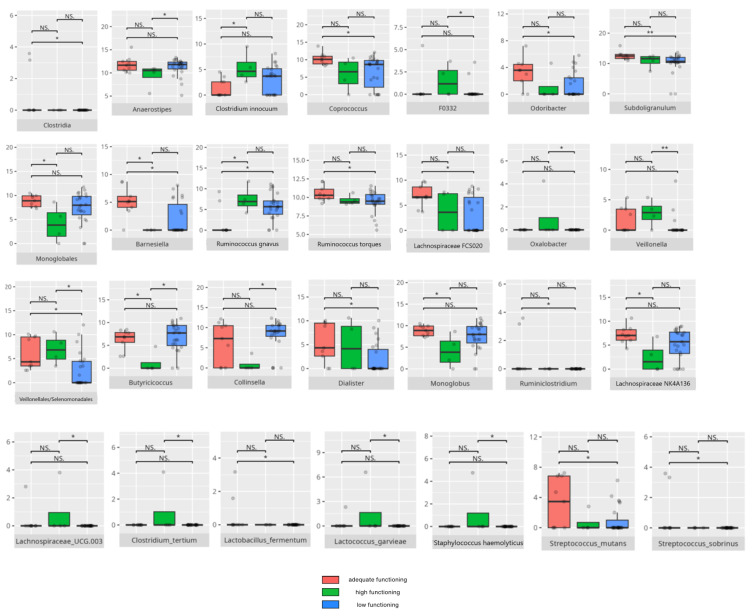
Statistically relevant differences between the two groups in the interpersonal skills subdomain (* *p* ≤ 0.05, ** *p* ≤ 0.01, NS: not significant).

The statistically important differences are detailed in Table 2 and illustrated in Figure 21.

##### Subdomain: Play and Leisure Skills

Five children (all boys) achieved high or moderately high scores in the play and leisure skills subdomain, while 30 children (25 boys and five girls) scored low or moderately low. Among the high scorers, three were school children, while two were adolescents. Two had been diagnosed with childhood autism. One presented with one or more FGIDs, and one had FS. All of them followed PBD and LCD. Of the lower-scoring group, 11 were preschoolers, 16 were school-aged children, and three were adolescents. Ten had diagnoses other than childhood autism. Thirteen presented with one or more FGIDs, and 13 had FS. The majority consumed PBD (n = 19) and HCD (n = 17).

**Figure 22:**
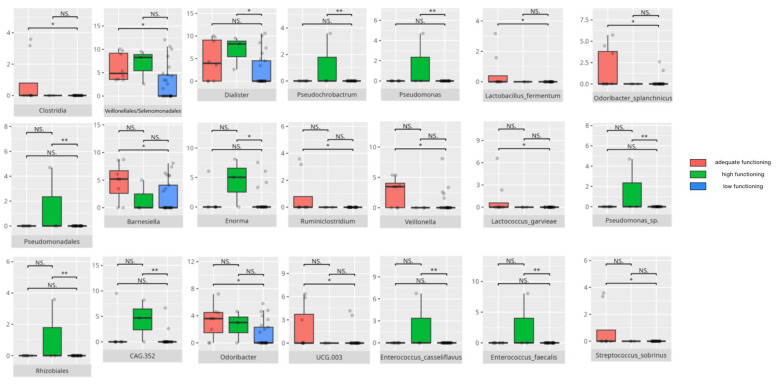
Statistically meaningful differences between the two groups in the play and leisure skills subdomain (* *p* ≤ 0.05, ** *p* ≤ 0.01, NS: not significant).

The statistically important differences are detailed in Table 2 and illustrated in Figure 22.

##### Subdomain: Coping Skills

**Figure 23:**
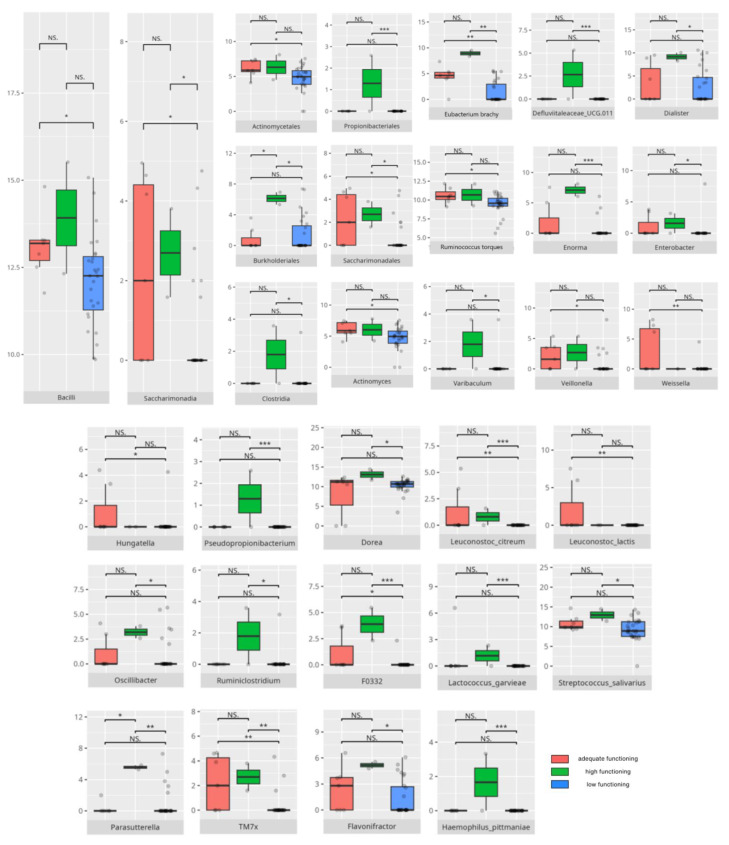
Statistically relevant differences between the two groups in the coping skills subdomain (* *p* ≤ 0.05, ** *p* ≤ 0.01, *** *p* ≤ 0.001, NS: not significant).

Four children scored high or moderately high in the coping skills subdomain, while 30 scored low or moderately low. Among the higher scorers, two were preschoolers and two were adolescents. All were boys. Two had been diagnosed with childhood autism. Two presented with one or more FGIDs, and one also had FS. All followed PBD, with three adhering to LCD. Of the lower-scoring participants, 10 were preschoolers, 17 were school-aged children, and three were adolescents. Twenty-four were boys. Nineteen were diagnosed with childhood autism. Thirteen had one or more FGIDs, and 12 had FS. Twenty-one participants consumed a PBD, while 17 followed an LCD. Statistically significant differences are detailed in Table 2 and illustrated in Figure 23. The V-score in coping skills showed a positive correlation with *F0332* abundance (r = 0.5). Table 3 contains a list of all correlations.

#### 3.5.4. Domain: Motor Skills

**Figure 24:**
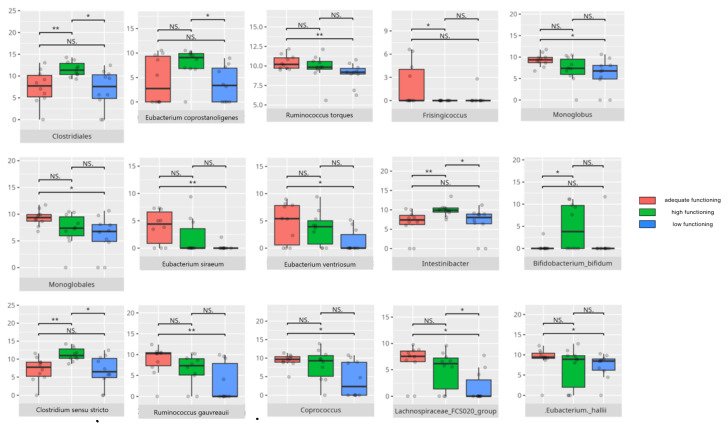
Statistically relevant differences between the two groups in the motor skills domain (* *p* ≤ 0.05, ** *p* ≤ 0.01, NS: not significant).

Thirteen children scored low or moderately low in the motor skills domain. Ten participants achieved an adequate result, while 13 scored high or moderately high. According to the Vineland Adaptive Behavioral Score scoring method, children aged 10 years or older were not assessed in the motor skills domain. The statistically important differences are detailed in Table 2 and illustrated in Figure 24.

##### Subdomain: Large Muscle Skills

**Figure 25:**
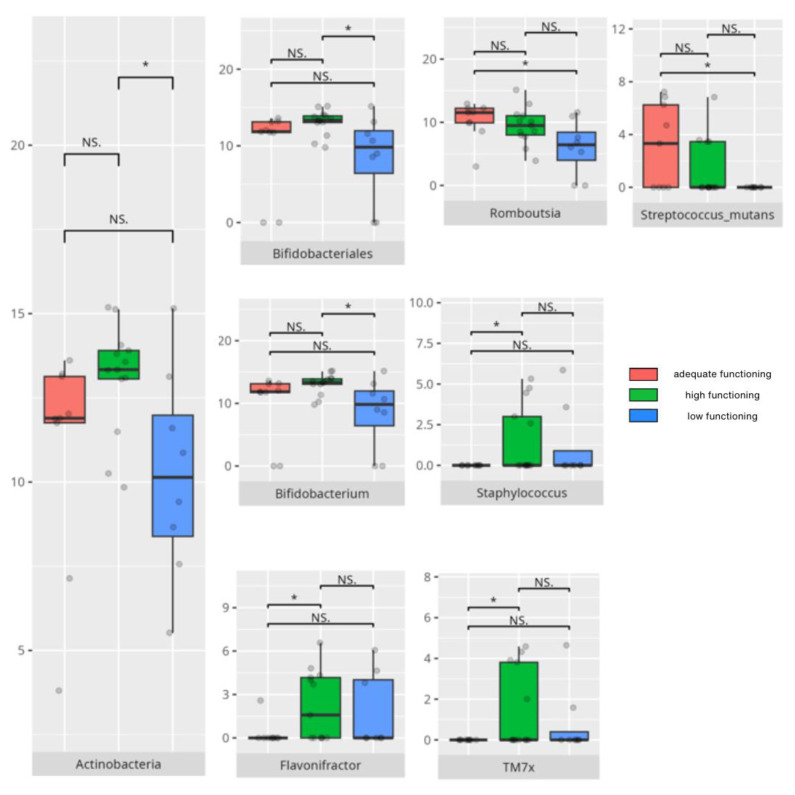
Statistically significant differences between the two groups in the large muscle skills subdomain (* *p* ≤ 0.05, NS: not significant).

Seventeen children (14 boys and three girls) scored high or moderately high in the large muscle skills subdomain, while 11 (eight boys and three girls) scored low or moderately low. Among the high scorers, six were preschoolers and 11 were school-aged children. Thirteen were diagnosed with childhood autism. Seven presented with one or more FGIDs, and seven had FS. Ten of these children followed PBD, and 13 adhered to HCD. In the lower-scoring group, six were preschoolers and five were school-aged children. Five had diagnoses other than childhood autism. Two presented with one or more FGIDs, and five had FS. The majority consumed PBD (n = 8) and HCD (n = 7). Statistically significant differences are detailed in Table 2 and illustrated in Figure 25.

##### Subdomain: Small Muscle Skills

**Figure 26:**
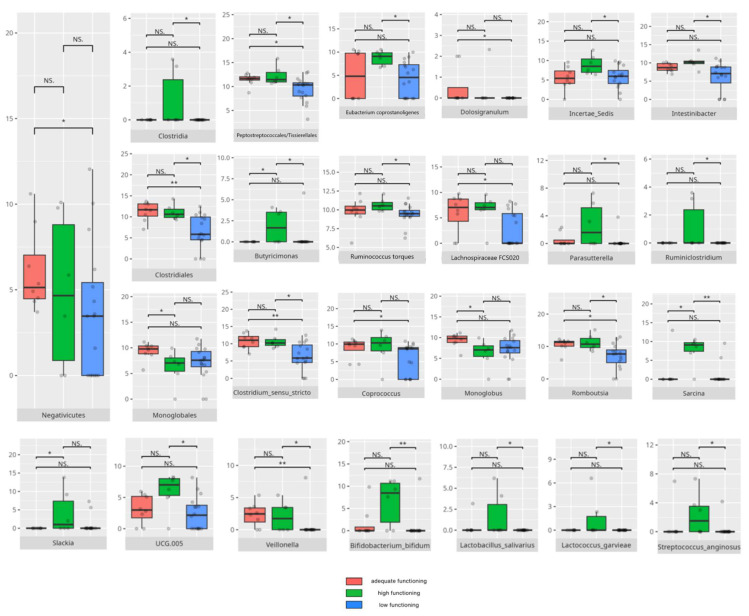
Statistically relevant differences between the two groups in the small muscle skills subdomain (* *p* ≤ 0.05, ** *p* ≤ 0.01, NS: not significant).

Eight children scored high or moderately high in the small muscle skills subdomain, while 20 scored low or moderately low. Among the higher scorers, one was a preschooler and seven were school-aged children. Six were boys, and six had been diagnosed with childhood autism. Two presented with one or more FGIDs, and three also had FS. Half of them followed LCD, and seven consumed PBD. Of the lower-scoring participants, eight were preschoolers and 12 were school-aged children. Sixteen were boys, and 14 had been diagnosed with childhood autism. Six presented with one or more FGIDs, while nine had FS. Eleven participants followed PBD, and 11 adhered to HCD. Statistically significant differences are detailed in Table 2 and illustrated in Figure 26.

The V-score in fine motor skills showed positive correlations with *Clostridiales* (r = 0.53) and *Peptostreptococcales/Tissierellales* (r = 0.57) abundance, as well as with *Romboutsia* abundance (r = 0.55). All correlations are summarized in Table 3.

## 4. Discussion

### 4.1. The Structure of the Study Group and Microbiota Composition

The composition of gut microbiota in individuals with ASD appears to be influenced by a variety of host-related factors, including age, sex, mode of delivery, diagnosis subtype, and dietary habits. The following analysis outlines key taxonomic differences associated with these variables, highlighting potential links between microbial profiles and clinical or neurodevelopmental characteristics.

Patients delivered via Cesarean section exhibited a higher relative abundance of *Clostridiales*, and at the genus level, *Clostridium sensu stricto*, which includes pathogenic species such as *Clostridium perfringens*—a microorganism previously implicated in post-Cesarean dysbiosis [23]. An increased prevalence of *Clostridiales* was also observed in the middle subgroup compared to older children, suggesting a less mature gut microbiota composition.

Notably, *Sellimonas*, a genus found to be elevated in individuals with schizophrenia, was also enriched in patients born via Cesarean section [24]. In contrast, vaginally delivered individuals showed higher levels of *Faecalitalea*, a genus known for short-chain fatty acid (SCFA) production and immunoregulatory properties [25]. These findings underscore the critical role of delivery mode in shaping early gut microbiota development.

*Micrococcales* and *Rothia*, both negatively correlated with age and recognized for their potential pathogenicity in humans [26,27,28], further support the hypothesis of microbiota immaturity in younger individuals.

Beyond *Clostridiales*, the middle subgroup demonstrated increased abundance of genera associated with neuropsychiatric conditions, including *Subdoligranulum*, which was previously found to be elevated in patients with major depressive disorder (MDD), and *Olsenella*, which has been linked to both MDD and insomnia [29,30,31]. Conversely, *Lachnospiraceae*, commonly reduced in Alzheimer’s disease (AD) [24], and *Romboutsia*, decreased in MDD [32], were found in lower abundance in this age group. *Adlercreutzia*, recognized for its anti-inflammatory properties [33], was also detected.

Adolescents presented with elevated levels of pathogenic taxa, such as *Klebsiella* and *Clostridium perfringens* [23,34], possibly reflective of their typical dietary patterns characterized by increased consumption of highly processed carbohydrate-rich foods and reduced fiber intake [35]. Nonetheless, their microbiota also showed greater representation of probiotic species [36,37,38], indicative of increased microbial maturity.

Sex-based differences were also apparent. *Peptococcales*, which were more abundant in boys, have been associated with mucin degradation [39], whereas girls exhibited higher levels of Rhodospirillales, known for their anti-inflammatory effects [40]. Other immunomodulatory taxa included *Eubacterium ruminantium* and *Lachnospiraceae UCG-010*, which—despite their regulatory roles—have also been associated with neurological and respiratory pathologies [41,42,43]. Similarly, *Ruminococcus gnavus*, enriched in males, and *Lautropia*, also predominantly found in boys, are taxa with known pathogenic potential [44,45].

In contrast, *Coprobacillus*, more prevalent in female participants, has been linked to a lower risk of irritable bowel syndrome (IBS) [46], while *Lachnospiraceae FCS020*, identified in boys, has been implicated in constipation among children with ASD [47]. *Odoribacter*, which has been described in neuropsychiatric microbiota profiles, was found to be reduced in Rett syndrome (RS) and increased in AD. Meanwhile, *Marvinbryantia*, which was more common in boys, has been reported to be decreased in AD [48]. *Lactobacillus fermentum*, which was detected across groups, is known for its anti-inflammatory properties and role in promoting neurological recovery post-stroke [49,50].

These findings suggest that although both sexes harbored microbial communities comprising taxa with pro- and anti-inflammatory as well as neuromodulatory potential, beneficial species appeared more frequently in girls, potentially contributing to sex-specific phenotypic presentations in ASD.

However, this divergence was not clearly reflected when comparing children diagnosed with childhood autism versus those with other neurodevelopmental disorders. Both diagnostic groups shared a proinflammatory microbiota profile [51,52,53,54]. Of note, *Ruminococcus torques*, predominantly observed in children with childhood autism, has shown ambiguous associations with cognitive impairment and dementia in prior studies [48,55,56].

### 4.2. The Influence of Diet on Gut Microbiota Composition

HCD was associated with an increased abundance of *Staphylococcales*, which is known for its pathogenic potential [57], and *Saccharimonadales*, which was previously observed to be elevated in patients with AD or mild cognitive impairment [58]. Similarly, *Anaerostipes* levels are elevated in Parkinson’s disease (PD) and RS [59,60]. In contrast, *Blautia* is decreased in MDD [61], while *Slackia*, noted for its anti-inflammatory properties, is reduced in lumbar degenerative spondylolisthesis [62]. *Weissella*, which is found in higher abundance among patients on LCD, is recognized for its beneficial health effects [63]. Notably, in our study, HCD was consumed by a majority of patients with food selectivity, consistent with the known tendency of individuals with ASD and food selectivity to experience weight abnormalities [64].

Among patients without food selectivity, *Peptostreptococcales/Tissierellales* were more abundant. This order has been described as potentially promoting increased cortical gray matter volume and was found in higher levels in individuals without insomnia or MDD symptoms [65]. Its abundance negatively correlated with the ratio of saturated fat to total fat intake, while positively correlating with complex carbohydrates consumption. Interestingly, both complex carbohydrate intake and the absence of food selectivity also correlated with *Clostridiales*, an order noted for its potential pathogenicity. *DTU089*, which was more prevalent in patients without food selectivity, was previously observed to decrease in patients with IBS following a starch- and sucrose-reduced diet [66]. Conversely, *Coprococcus* has been associated with individuals not experiencing or experiencing mild IBS symptoms [46]. *Barnesiella*, which decreased in RS [24], and *Eubacterium coprostanoligenes*, which has been linked to cognitive impairment unrelated to AD and colorectal cancer risk, were also noted [67,68]. *Lachnospiraceae NK4A136*, which has been reported to have a causal association with puerperal sepsis [69], contrasted with *Turicibacter*, which exerted potentially beneficial effects on lipid and bile acids metabolism in a murine model [70]. These findings imply that food selectivity may not be a primary driver of overall microbiota composition.

*Eubacterium coprostanoligenes*, which negatively correlates with saturated fat intake, demonstrated a hypocholesterolemic effect in a rabbit model [71]. *Eubacterium hallii* similarly showed a negative correlation with total cholesterol level in humans and improved insulin sensitivity in mice [72,73]. *Dorea*, which is more abundant in PD, multiple sclerosis (MS), and IBS patients [74,75], is also elevated in overweight and obese women consuming moderately high protein diets [76]. *Leuconostoc citreum* and *Leuconostoc lactis*, which are positively correlated with protein intake, are recognized for their anti-inflammatory and antibacterial properties [77,78]. In contrast, saturated fat consumption correlated positively with species known for proinflammatory and pro-oncogenic activity [52,53,57,79,80]. Notably, *Dielma* showed a positive correlation with the ratio of unsaturated fat to total fat intake, mean fat consumption relative to weight, and high-protein diet. This genus is elevated in patients with post-stroke sleep disturbance [81], although it decreases in male adults on high-protein diets [76] and is reduced in cases of malnutrition associated with esophageal neoplasms [82].

Patients with high protein intake also had elevated levels of *Eubacterium brachy*, a genus linked to exacerbation of atopic dermatitis [83] but paradoxically associated with a lower risk of insomnia characterized by difficulty falling asleep [31]. Additionally, *Lactobacillus curvatus*, described as neuroimmune modulator, reduces the risk of age-related memory deficits in a murine model [61].

Higher consumption of simple carbohydrates and the proportion of energy derived from them correlated positively with *Epulopiscium*, which has been previously linked to impaired cognitive functions [84], as well as with *Weissella*, a proposed probiotic genus. Simple carbohydrate intake correlated negatively with *Flavonifractor*, a genus known to induce oxidative stress and which is more abundant in bipolar disorder (BD) patients [24,85]. Energy from carbohydrates also showed a positive correlation with *Klebsiella*, recognized for its pro-inflammatory characteristics [34], while total kilocalorie intake from carbohydrates correlated with *Clostridium perfingens*, a known agent of intestinal diseases [86].

### 4.3. The Role of Gut Microbiota in Functional Gastrointestinal Disorders

The occurrence of FGID symptoms in patients with ASD represents a significant clinical challenge, as it substantially lowers quality of life—not only due to the symptoms themselves, but also because of associated social stigma, depression, and anxiety [87,88]. Several bacteria genera that were found to be more abundant in ASD patients, including *Oscillibacter*, *Holdemania*, *Eubacterium coprostanoligenes*, or *Enterobacter*, have also been linked to MDD [30]. *Oscillibacter* is reduced in RS, while *Lachnospira* is decreased in AD [24,89]. *Papillibacter*, which correlates positively with Mini-Mental Test Examination scores and negatively with neuropsychiatric symptoms of AD [90], is less abundant in these patients but increased in PD [91]. *Dielma* has been observed at elevated levels in individuals experiencing post-stroke sleep disturbances [81]. Importantly, neuropsychiatric disorders can exacerbate gastrointestinal symptoms.

*Lachnospiraceae UCG.004* has been described as being protective against gastroesophageal reflux disease [92]. Although exopolysaccharides produced by *Leuconostoc mesenteroides* partially convert to SCFA, which is beneficial for intestinal health, certain polysaccharides like dextran may contribute to FGID through their osmotic effects [93].

*Staphylococcus* has been implicated in constipation, particularly among individuals consuming high-protein and high-fat diets [94]; however, some evidence suggests that early-life colonization with *Staphylococcus* may reduce constipation risk in infants [95]. *Clostridium innocuum*, despite its known association with diarrhea [96], was found to be more abundant in children experiencing constipation. Moreover, *Eubacterium hallii*, which has been postulated as a next-generation probiotic, was also more abundant in the individuals with constipation and bloating [97]. In contrast, SCFA-producing bacteria such as *Agathobacter* and *Lactococcus lactis* were more common in children without constipation. *Agathobacter* is depleted in Crohn’s disease, suggesting anti-inflammatory influence [98], while *Lactococcus lactis* has demonstrated efficacy as a probiotic for mild constipation in adults [99].

*Oxalobacter*, which is more abundant in patients without functional diarrhea or bloating, has a role in oxalate degradation—a key factor in kidney stone formation [100]—though the relationship between oxalate and diarrhea remains unclear. Conversely, *Enterobacter* contributes to FGID symptoms through hydrogen sulfide production in the colon, which metabolizes into sulfuric acid, damaging cells and causing immune, secretory, and motility disturbances that increase visceral sensitivity [101], potentially leading to diarrhea. *Family XII UCG.001* has been associated with neuroendocrine neoplasms [102]. While *Leuconostoc lactis* and *Leuconostoc citreum* exhibit anti-inflammatory and antibacterial properties [77,78], their abundance was paradoxically higher in participants with diarrhea. Interestingly, individuals without diarrhea showed higher levels of *Enterobacter cloacae*, a species known for its proinflammatory effects [52,53]. *Lactobacillus brevis*, which has been used successfully as a probiotic to reduce the pro-inflammatory activity of several bacteria species [37,103], also produces γ-aminobutyric acid (GABA), a crucial inhibitory neurotransmitter involved in neuropsychiatric disorders [61]. By contrast, *Lactobacillus oligofermentans* has not yet been characterized for its probiotic potential.

The *Bacilli* class, observed in greater abundance in participants without bloating, includes both pathogenic species such as *Bacillus cereus* and species with probiotic properties that alleviate gastrointestinal symptoms [104]. This class is typically elevated in children with ASD [105]. *Eubacterium eligens*, on the other hand, is usually reduced in ASD patients compared to typically developing ones [106]. Both species that were more abundant in children without bloating are recognized for their probiotic potential [36,107]. Additionally, *Lactobacillus delbrueckii* has been proposed as a psychobiotic candidate due to its anxiolytic activity, as demonstrated in a fish model [108]. *Eubacterium coprostanoligenes*, found in higher levels in participants reporting bloating, has been previously linked with gastrointestinal symptoms in ASD patients.

### 4.4. The Influence of Microbiota Composition on Communication Skills

Communication skills in patients showed strong associations with the abundance of SCFA producers and neuroimmune modulators, whereas lower communication scores related to higher levels of proinflammatory and potentially pathogenic bacteria.

*Proteobacteria*, which are generally elevated in individuals with ASD [8], were observed in higher-functioning patients. Similarly, improved communication abilities, particularly in the receptive domain, were linked to increased levels of SCFA-producing bacteria such as those in the *Bacilli* class [109]. SCFAs are known to regulate neuroimmune responses in the central nervous system (CNS), potentially reducing neuroinflammation and protecting against neurodegeneration. Additionally, some *Bacilli* members modulate neurodevelopment by influencing neurotransmitter production [110]. The receptive communication subdomain was also positively associated with *Veillonellales/Selenomonadales*, another SCFA-producing group whose role in ASD remains ambiguous; some studies have reported decreased abundance in ASD children, while others found increased levels in those with severe symptoms [60,111].

Interestingly, patients with better communication scores also had elevated levels of other SCFA producers with mixed impacts on health and neuromodulation. For instance, *Peptostreptococcales/Tissierellales*, which can be harmful, are abundant in PD and in individuals under chronic stress [24], while *Tyzzerella* is elevated in MS [112]. *Flavonifractor*, enriched in children with high expressive and receptive communication skills and positively linked to receptive abilities, is known to induce oxidative stress and is elevated in schizophrenia, MDD, and BD [24,86]. Yet, it may have protective effects in vascular diseases by reducing arterial stiffness [113]. *Eisenbergiella* and *Hungatella*, also increased here, are linked to MS [114]; Hungatella’s association with depressive symptoms is thought to involve stimulating GABA production [29]. On the other hand, its abundance is elevated in infants succeeding at the Point and Gaze test, indicative of further language development [115].

The genus *Enterococcus* has demonstrated a positive influence on infant neural rhythm tracking—an important factor in communication development [115]—and was found reduced in ASD but elevated in PD and RS [24]. Other genera noted include *Enorma* (increased in spinal muscular atrophy (SMA)), *Anaerococcus* (elevated in schizophrenia), and *Gemella* (typically decreased in ASD) [116,117]. Among receptive communication high scorers, pro-inflammatory genera like *Dialister* and *CAG.352*, and pro-allergic *GCA.90006657* were more abundant [51,62]. *Intestinibacter*, which is potentially harmful, has been linked to neurodevelopmental disorders [118]. Higher expressive communication was associated with increased *Eubacterium* abundance. While *Eubacterium coprostanoligenes* is pro-inflammatory that exacerbates gastrointestinal symptoms in ASD, *Eubacterium brachy* appears beneficial, alleviating insomnia [31,119].

*Bifidobacterium animalis* stands out as a neuroimmunomodulatory species, showing benefits in neurological conditions, including ASD murine models [120,121]. *Lactococcus garvieae* enhances intestinal mucosal integrity [122], and *Streptococcus salivarius* produces bacteriocins contributing to antimicrobial activity [123].

*Oscillospirales*, which were more abundant in children with adequate expressive communication, shows an ambiguous role in the CNS, i.e., they are decreased in PD and RS but increased in anti-N-methyl-D-aspartate (anti-NMDAR) encephalitis and in male rodent models of autism [124,125,126]. *Ruminiclostridium*—a SCFA producer linked to alleviating rhythm disruptions [127,128]—was enriched in adequate and high-scoring patients in communication subdomains. Conversely, *Dielma*, elevated in individuals with post-stroke sleep disorders [81], was linked to adequate communication scores but lower writing skills. *Phascolarctobacterium* is typically higher in ASD [8], while *Lachnospiraceae*, another SCFA producer, is increased in AD [89]. *Paraprevotella* was positively associated with cognitive functioning in AD [129]. *Catenisphaera*, which was more abundant in adequate writing scorers, is decreased in violent women [130].

*Oscillibacter*, elevated in adequate-scoring children, was reduced in RS but increased in MDD and is especially related to negative affect [24,30,131]. MDD patients also show increased *Faecalibacterium*—an SCFA producer linked to anti-inflammatory effects—whose abundance is reduced in RS, AD, multi-system atrophy (MSA), PD, MS, and schizophrenia [24,132]. *Romboutsia*, considered pro-inflammatory, is paradoxically decreased in MDD [32,133]. *Faecalitalea*, which was more abundant in adequate receptive communicators, produces SCFAs but may increase in inflammatory bowel diseases, indicating a complex role in gut homeostasis [25,134]. *Dolosigranulum*, which was elevated in those with adequate expressive skills, has potential probiotic properties [135].

*Leuconostoc* species, known for immunomodulatory capacity, were more abundant across adequate scoring groups [77,78,136]. In writing subdomain adequate scorers, *Lactobacillus fermentum*—an SCFA producer—alleviates gastrointestinal inflammation and post-stroke neurological symptoms [49,50]. *Bifidobacterium bifidum* may impede neurodegeneration progression [128], while *Streptococcus sobrinus*, although pro-inflammatory in oral health, is less characteristic in gut microbiota [137].

*Clostridia*, associated with lower overall communication functioning, are elevated in ASD—especially in severe cases—and in RS, MSA, and MDD, though reduced in PD and MS [8,24]. Notably, *Clostridia* was also positively associated with neural rhythm tracking [115], possibly explaining their higher abundance in those with better receptive and expressive scores.

Other bacteria linked to lower communication skills often include opportunistic or proinflammatory species that are capable of disrupting neural signaling and acting as pathobionts [24,138,139,140,141,142,143]. Their ability to trigger systemic inflammation may impair neurodevelopment and communication. *Clostridium*, *Pseudomonas*, *Enterococcus*, and *Haemophilus* species enriched in low scorers have known proinflammatory and infection-related roles [140,144,145,146,147]. Intriguingly, *Rhizobiales*, *Pseudomonadales*, and *Enterococcus* were more abundant in children with adequate writing skills, despite being linked to lower overall communication scores; *Enterococcus* notably also participates in MS pathophysiology [147]. *Erysipelotrichaceae UCG.003*, which is associated with Western diet consumption, was elevated in RS [24,96], though some taxa like *Defluviitaleaceae UCG-011* were decreased in MDD [61]. *Collinsella*, which was more abundant in poorer writing performers, was elevated in ASD [66], whereas *Blautia* was decreased in MDD [61]. *Ruminococcus gauvreauii*, a SCFA producer linked to prosocial behavior [148], was increased in low-writing scorers. *Anaerostipes*, which was negatively correlated with writing skills, was elevated in PD and RS [59,60].

### 4.5. Microbiota Composition and Daily Living Skills

Daily living skills exhibited a complex pattern of associations with both pro- and anti-inflammatory bacteria, alongside genera linked to neuropsychiatric disorders.

*Gammaproteobacteria*, which has been found in higher abundance among patients with strong daily living skills—including domestic ones—have also been reported as elevated in PD [149]. *Negativicutes*, which are more prevalent in patients with adequate scores compared to low scorers, were previously observed predominantly in typically developing children versus those with ASD [150].

Interestingly, *Lactobacillales*, known for their probiotic activity [37,38], were more abundant in low-scoring patients, including those with reduced personal and domestic skills. *Propionibacteriales*, which were higher in children with adequate personal skills than in those with higher skills, are recognized for their potential to cause infections [139].

Lower scores in daily living skills were linked to an increased abundance of pro-inflammatory bacteria such as *Pseudomonas*, *Ruminiclostridium*, *Granulicatella*, and *Parvimonas* [140,151,152]. Additionally, these patients exhibited higher levels of *Oxalobacter*—a genus involved in oxalate degradation [100]—and *Anaerostipes*, *Collinsella*, and *Butyricicoccus*, genera linked to various neurological conditions, such as PD, MD, RS, and schizophrenia [24,59,60,74]. Moreover, *Collinsella* and *Ruminococcus* were previously observed to be more abundant in children with ASD [60], with the latter being simultaneously decreased in RS [24]. Most species associated with low scoring were pro-inflammatory, except for *Lactococcus garvieae*, *Lactobacillus fermentum*, and *Odoribacter splanchnicus*, which have anti-inflammatory or probiotic properties despite being abundant in children with poorer domestic skills [50,122,153].

Adequate daily living skill scores were associated with a mix of pro-inflammatory genera—such as *Ruminiclostridium*, *Sarcina*, *Staphylococcus*, *Eubacterium siraeum*, *Haemophilus*, and *NK4A214* [57,69,140,154,155,156]—and genera prevalent in neurological disorders, including *Anaerostipes* [59]. Simultaneously, beneficial genera like *Dolosigranulum*, *Butyricimonas*, *Agathobacter*, and *Coprococcus* were also more abundant [46,98,135,157]. Some taxa, like *Oscillibacter* showed mixed influence; while being reduced in RS and known for anti-inflammatory effects in the gut, they were elevated in MDD [24,30,46]. *Clostridium sensu stricto* contains both beneficial and harmful strains and exhibits altered abundance in neuropsychiatric disorders [24]. *Lachnospiraceae*, noted for anti-oncogenic intestinal effects and decreased in PD, was paradoxically correlated with constipation in ASD [47,89,158]. *Barnesiella* and *Odoribacter*, which were more abundant in children with adequate community skills scores, were decreased in RS [24], while *Parabacteroides* levels were reduced in MS but increased in PD [74]. *Alistipes*, which was more abundant in adequate scorers in community skills, is typically reduced in children with ASD [66]. Most species elevated in adequate community skills were anti-inflammatory [50,123,153], with the exception of *Streptococcus sobrinus* [137].

Higher daily living skill scores were also linked to several pro-inflammatory genera, including *Dialister*, *CAG.352*, *Intestinibacter*, *Romboutsia*, *Flavonifractor*, *Senegalimassilia*, *Colidextribacter*, and *Escherichia/Shigella*; the latter was also increased in RS and associated with anxiety [24,62,85,133,159,160,161,162]. *Romboutsia* was decreased in MDD [32], whereas *Colidextribacter* was reduced in PD [163]. Interestingly, despite its pro-inflammatory nature, *Intestinibacter* positively correlated with domestic skills, and *Romboutsia* with personal skills. Children with stronger personal skills showed higher abundances of *Eubacterium brachy*—known to alleviate insomnia—and *Eubacterium coprostanoligenes*, considered pro-inflammatory [31,119]. *GCA.900066575* appears to have an anti-inflammatory role in food allergies [51].

Several bacterial genera that were more abundant in participants with higher daily living skills scores have likewise been associated with other neuropsychiatric disorders. *Tyzzerella* has been associated with MS progression and psychoneurological symptoms in head and neck cancer patients [112,164]. *Papillibacter* is decreased in AD but elevated in PD [90,91], while *Anaerococcus* is more abundant in schizophrenia [117]. *Veillonella*, which was enriched in those with higher personal skills, is typically decreased in children with ASD [150]. *Enterococcus*, previously discussed, is linked to improved neural rhythm tracking and was elevated in RS patients but reduced in ASD [24,114]. In contrast, *Hungatella*, connected to MDD and MS, was found to be decreased in children who scored higher on the Point and Gaze test, which measures joint attention [29,114,115].

### 4.6. The Role of Microbiota in the Development of Socialization Skills

The socialization domain showed associations with both pro- and anti-inflammatory bacteria.

Patients with lower socialization scores had higher abundances of *Clostridia* and *Desulfovibrionales*, taxa previously linked to more severe ASD symptoms [8,61]. *Clostridia* were also elevated in RS, MSA, and MDD [24]. Low scorers in the interpersonal skills subdomain exhibited increased levels of pro-inflammatory genera such as *Ruminiclostridium*, as well as bacteria commonly found in neurological disorders, including *Anaerostipes*, *Butyricicoccus*, and *Collinsella* [24,59,74]. In contrast, *Barnesiella*, which was reduced in RS [24], was lower in these low-scoring patients. Interestingly, both poorer and adequate performers showed higher levels of *Lactococcus garvieae* and *Lactobacillus fermentum*, which protect intestinal mucosa and promote SCFA-mediated recovery post-stroke [51,122], alongside a paradoxical increase in the pro-inflammatory *Streptococcus sobrinus* [165].

Children with slightly better socialization outcomes, scoring in the adequate range, had increased abundances of potentially pathogenic *Enterobacterales* [101,166], as well as *Veillonellales/Selenomonadales*, which are typically decreased in patients with ASD [150]. Adequate scorers in coping skills also had elevated *Bacilli*, known to produce neurotransmitters such as dopamine and noradrenaline, a bacterial class typically increased in ASD [105,110]. These individuals additionally showed higher levels of *Saccharimonadia*, associated with cognitive impairments [58], and more of the potentially protective *Monoglobales* compared to high scorers [167].

At the genus level, adequate scorers displayed higher abundances of the pro-inflammatory *Dialister* alongside the anti-inflammatory *Coprococcus* [46,62]. *Enterococcus*, which has been linked to improved neural rhythm tracking and was typically decreased in ASD but elevated in PD and RS, was also more abundant [24,115]. *Subdoligranulum*, associated with MDD, and *Hungatella*—linked to depression and also elevated in these adequate scorers in coping skills—were noted [29]. Other genera enriched in this group included *Eubacterium brachy*, which is known for reducing insomnia risk, and *Weissella*, which has been recognized for its probiotic potential [31,63]. *Ruminococcus torques*, which was more abundant in children with adequate interpersonal and coping skills, was increased in PD and ASD but decreased in Alzheimer’s disease (AD) [48,58,168]. *Veillonella*, which was higher in adequate-scorers in the play and leisure and in the coping skills subdomains, and *Actinomyces*, which were higher in adequate-performers in the coping skills subdomain, are typically decreased in children with ASD [150]. *Butyricicoccus* was linked to MS and PD [74]. *Odoribacter* was previously observed to be decreased in RS [24], with *O. splanchnicus*, which was observed in adequate scorers in the play and leisure skills subdomain, known for its probiotic potential [153]. Children with adequate coping skills also carried more immunomodulatory *Leuconostoc citreum* and *Leuconostoc lactis* [77,78,136]. However, adequate scorers in the interpersonal and the play and leisure skills also carried pro-inflammatory *Streptococcus mutans* and *Streptococcus sobrinus* [169].

High-scoring children in socialization exhibited greater abundances of *Bilophila*, which is typically reduced in ASD but raised in MS and schizophrenia, *Olsenella*, which is increased in MDD, and *Oscillibacter*, which is decreased in RS [24,30,60]. *Clostridia*, which was elevated in high scorers in coping skills and linked to severe ASD symptoms as well as other neuropsychiatric disorders, showed positive associations with neural rhythm tracking [24,115]. *Burkholderiales*, which is known to be more abundant in ASD, and *Defluviivitaleaceae*, which is decreased in MDD, were also observed [170,171]. *Oscillibacter* appeared to be linked to MDD but was reduced in RS [24,30], while *Dorea* was more abundant in MS and PD [74]. Several pro-inflammatory genera—including *Parasutterella*, *Ruminococcus gnavus*, *CAG.352*, *Rhizobiales*, *Pseudomonadales*, *Eubacterium brachy*, *Dialister*, *Enterobacter*, *Flavonifractor*, *Varibaculum*, and *Propionibacteriales*—were elevated [24,44,52,53,62,83,85,139,140,141,172,173]. *Lachnospiraceae* and *Clostridium innocuum* demonstrated ambiguous roles in neurological disorders and inflammation [47,91,96,158], whereas the significance of *Oxalobacter* in ASD remains unclear [100]. *Ruminiclostridium*, which produces beneficial SCFAs and helps alleviate rhythm disruptions, was also noted [127,128]. Overall, higher socialization scores were linked to greater abundances of several pro-inflammatory species [144,145,146,156,174], except for *Lactococcus garvieae*, *Streptococcus salivarius*, and *Leuconostoc citreum*, all of which are associated with anti-inflammatory or probiotic functions [78,122,175].

### 4.7. Microbiota and the Development of Motor Skills

Motor skills showed associations with both protective and pro-inflammatory bacteria, highlighting the complex role of microbiota in neurological function.

The *Monoglobales* order, known for its protective potential, was more abundant in patients with adequate rather than high scores in the motor skills domain [167]. Adequate fine motor skills were linked to higher levels of *Negativicutes*, a group previously found to be reduced in ASD [150]. Enhanced fine motor performance was also associated with increased abundance of *Peptostreptococcales/Tissierellales*, an order connected to greater cortical gray matter volume and absence of insomnia or depression [65], which may be important for motor development. At the genus level, children exhibiting better motor skills showed elevated levels of protective bacteria such as *Ruminococcus gauvreauii*, *Coprococcus*, and *Eubacterium hallii* [46,97,148]. However, some pro-inflammatory taxa, like *Eubacterium siraeum* [155], were also more abundant in this group. Intriguingly, certain genera with protective attributes had conflicting associations with neuropsychiatric disorders; for example, *Eubacterium ventriosum* was increased in MDD but decreased in AD [29,48]. Similarly, *Lachnospiraceae* and *Ruminococcus torques* were reduced in AD, while *R. torques* was elevated in ASD [48,168]. *Romboutsia*, a pro-inflammatory genus elevated in adequate-functioning patients with gross motor skills and in high-functioning patients with fine motor skills, is known to be increased in MDD [32,133]. Additionally, *Frisingicoccus* negatively correlated with anxiety and motor deficits in a PD rodent model and was elevated in SMA [116,157].

Patients with high motor skills scores exhibited greater abundances of anti-inflammatory bacteria, including *Eubacterium coprostanoligenes* and members of *Bifidobacteriales*—especially *Bifidobacterium bifidum* [119,176]—as well as pro-inflammatory *Intestinibacter* [118]. Notably, high-functioning individuals, particularly those with superior fine motor skills, correlated with increased *Clostridiales* abundance. While *Clostridiales* were linked to more severe ASD symptoms and dysbiosis in RS, MSA, and MDD, they were also associated with enhanced neural rhythm tracking, a mechanism essential for fine motor skill development [8,24,61,115]. Fine motor abilities further correlated with higher levels of anti-inflammatory taxa, such as *Butyricimonas*, *Lactobacillus salivarius*, *Lactococcus garvieae*, and *Bifidobacterium bifidum* [122,123,157,176], as well as SCFA-producing bacteria, such as *Ruminiclostridia* [127,128]. Simultaneously, several pro-inflammatory bacteria—including *Eubacterium coprostanoligenes*, *Parasutterella*, *Romboutsia*, *Intestinibacter*, and *Streptococcus anginosus* [31,118,133,172,177]—were enriched among individuals excelling in fine motor skills, alongside neuropsychiatric disorder-associated genera like *Ruminococcus torques* and *Sarcina* [55,168,178].

Interestingly, gross motor skills appeared to improve with increased abundances of pro-inflammatory taxa such as *Flavonifractor* and *Staphylococcus* [24,57,85], with the exception of *Bifidobacterium*, which is generally protective [176]. *TM7x*, notable for its highly reduced genome and parasitic lifestyle on other bacteria [179], was also more abundant in patients exhibiting better gross motor function, illustrating the intricate microbial interactions influencing host physiology.

### 4.8. Limitations

This study offers new understanding of the intricate relationship between gut microbiota and behavioral, dietary, and gastrointestinal factors in children with ASD. However, some limitations must be considered. First, the cross-sectional nature of the study prevented establishing any cause-and-effect relationship between microbial composition and clinical characteristics. Long-term studies are necessary to clarify whether the microbial patterns observed are a result or a cause of ASD-related symptoms. Second, despite careful selection of the cohort, the sample size was relatively small, especially for subgroup analyses based on sex, age, diet, or adaptive functioning. This constraint may affect the broader applicability of the results and highlights the importance of validating these findings in larger and more diverse groups.

## 5. Conclusions

This study highlights the significant influence of delivery mode, diet, age, and sex on gut microbiota composition in children with neurodevelopmental and neuropsychiatric disorders. The complex interplay between microbial communities and clinical symptoms—such as communication, motor skills, socialization, and gastrointestinal function—suggests a strong gut–brain–immune connection. Both pro-inflammatory and anti-inflammatory bacterial genera contribute variably to neurological outcomes, indicating that microbial balance is critical in symptom severity and development. These findings underscore the importance of considering microbiota-targeted interventions as part of comprehensive care strategies for affected individuals.

## Figures and Tables

**Table 1:** Detailed data analysis of the structure of the group, diet, and functional gastrointestinal disorders (FGID).

	Total Number of Participants: n = 45	
Sex	Female	n = 7
	Male	n = 38
Age (distribution: 7.9 ± 3.35)	2–6 years	n = 12
	6–14 years	n = 28
	15–18 years	n = 5
Diagnosis according to ICD-10	Childhood autism	n = 30 (67%)
	Other diagnosis	n = 15 (33%)
Mode of delivery	Vaginal delivery	n = 31 (69%)
	Cesarean section	n = 14 (31%)
Gastrointestinal symptoms	The presence of any FGID	n = 18 (40%)
	Functional constipation	n = 8 (18%)
	Functional diarrhea	n = 9 (20%)
	Functional bloating	n = 7 (16%)
Diet	Type of diet (macroelements)	Protein-based: n = 31 (6%)
		Carbohydrate-based: n = 14 (31%)
	Type of diet (calorie intake)	Low-calorie diet: n = 23 (51%)
		High-calorie diet: n = 12 (49%)
	Food selectivity	n = 16 (36%)
	Mean daily protein consumption (g/day)	90.05 ± 31.06
	Mean protein intake normalized to body weight (g/kg BW)	3.81 ± 1.71
	Mean daily carbohydrate consumption (g/day)	251.02 ± 62.66
	Percentage of energy from carbohydrates (%)	51.41 ± 9.31
	Mean proportion of simple carbohydrates in total carbohydrate intake (%)	41.31 ± 7.82
	Mean daily fat consumption (g/day)	79.51 ± 31.32
	Mean fat intake normalized to body weight (g/kg BW)	3.3 ± 1.7
	Fraction of unsaturated fatty acids within total fat intake (%)	59.89 ± 5.30

**Table 2:** Differences in gut microbiota across patient groups.

Feature	Bacteria	*p*-Value ≤
Class		
high-calorie diet	*Saccharimonadia*	0.05
no bloating	*Bacilli*	0.05
communication—expressive skills (adequate > low)	*Clostridia*	0.05
communication—expressive skills (high > low)	*Negativicutes*	0.05
communication—writing skills (low > high)	*Coriobacteria*	0.05
communication—total (high > adequate)	*Bacilli*	0.05
communication—total (high > low)	*Alphaproteobacteria*	0.05
	*Gammaproteobacteria*	0.05
daily living skills—domestic skills (adequate > low)	*Negativicutes*	0.05
daily living skills—domestic skills (low > high)	*Gammaproteobacteria*	0.05
daily living skills—community skills (adequate > low)	*Negativicutes*	0.01
daily living skills—total (high > low)	*Gammaproteobacteria*	0.05
daily living skills—total (high > adequate)	*Gammaproteobacteria*	0.05
socialization—coping skills (adequate > low)	*Bacilli*	0.05
	*Saccharimonadia*	0.05
socialization—coping skills (high > low)	*Saccharimonadia*	0.05
using large muscles (high > low)	*Actinobacteria*	0.05
using small muscles (adequate > low)	*Negativicutes*	0.05
Order		
age (school children > adolescents)	*Clostridiales*	0.05
sex: female	*Rhodospirillales*	0.05
sex: male	*Peptococcales*	0.05
Caesarean’s section	*Clostridiales*	0.05
high-calorie diet	*Saccharimonadales*	0.05
	*Staphylococcales*	0.01
no food selectivity	*Peptostreptococcales/Tissierellales*	0.05
communication—expressive skills (adequate > low)	*Clostridiales*	0.001
	*Oscillospirales*	0.05
	*Peptostreptococcales/Tissierellales*	0.05
communication—expressive skills (high > low)	*Clostridia*	0.05
	*Peptostreptococcales/Tissierellales*	0.01
	*Veillonellales/Selenomonadales*	0.05
communication—receptive skills (high > low)	*Clostridia*	0.01
	*Peptostreptococcales/Tissierellales*	0.05
communication—receptive skills (high > adequate)	*Veillonellales/Selenomonadales*	0.05
communication—writing skills (low > high)	*Coriobacteriales*	0.05
communication—writing skills (low > adequate)	*Lachnospirales*	0.05
communication—writing skills (adequate > low)	*Pseudomonadales*	0.001
	*Rhizobiales*	0.001
communication—total (low > adequate)	*Actinomycetales*	0.05
	*Lactobacillales*	0.05
communication—total (low > high)	*Clostridia*	0.05
	*Propionibacteriales*	0.05
	*Pseudomonadales*	0.05
	*Rhizobiales*	0.05
communication—total (adequate > low)	*Acidaminococcales*	0.05
	*Clostridia*	0.05
communication—total (adequate > high)	*Acidaminococcales*	0.05
communication—total (high > low)	*Peptostreptococcales/Tissierellales*	0.05
communication—total (high > adequate)	*Lactobacillales*	0.05
daily living skills—personal skills (adequate > low)	*Clostridiales*	0.01
daily living skills—personal skills (high > low)	*Enterobacterales*	0.05
	*Peptostreptococcales/Tissierellales*	0.01
	*Veillonellales/Selenomonadales*	0.05
daily living skills—domestic skills (low > adequate)	*Clostridia*	0.05
Clostridia	*Lachnospirales*	0.05
	*Pseudomonadales*	0.05
	*Rhizobiales*	0.05
daily living skills—domestic skills (adequate > low)	*Clostridiales*	0.01
	*Veillonellales/Selenomonadales*	0.05
daily living skills—domestic skills (high > low)	*Enterobacterales*	0.05
	*Peptostreptococcales/Tissierellales*	0.05
	*Veillonellales/Selenomonadales*	0.05
daily living skills—community skills (adequate > low)	*Clostridia*	0.01
	*Veillonellales/Selenomonadales*	0.05
daily living skills—community skills (adequate > high)	*Pasteurellales*	0.05
daily living skills—community skills (high > low)	*Enterobacterales*	0.05
daily living skills—total (low > high)	*Lachnospirales*	0.05
	*Pseudomonadales*	0.05
	*Rhizobiales*	0.05
daily living skills—total (adequate > low)	*Clostridia*	0.05
	*Clostridiales*	0.05
daily living skills—total (high > low)	*Enterobacterales*	0.05
	*Peptostreptococcales/Tissierellales*	0.05
	*Veillonellales/Selenomonadales*	0.05
socialization—interpersonal skills (low > adequate)	*Clostridia*	0.05
socialization—interpersonal skills (adequate > low)	*Veillonellales/Selenomonadales*	0.05
socialization—interpersonal skills (adequate > high)	*Monoglobales*	0.05
socialization—interpersonal skills (high > low)	*Veillonellales/Selenomonadales*	0.05
socialization—play and leisure skills (adequate > low)	*Clostridia*	0.05
	*Veillonellales/Selenomonadales*	0.05
socialization—play and leisure skills (high > low)	*Pseudomonadales*	0.01
	*Rhizobiales*	0.01
socialization—coping skills (adequate > low)	*Actinomycetales*	0.05
	*Saccharimonadales*	0.05
socialization—coping skills (high > low)	*Burkholderiales*	0.05
	*Clostridia*	0.05
	*Propionibacteriales*	0.001
	*Saccharimonadales*	0.05
socialization—coping skills (high > adequate)	*Burkholderiales*	0.05
socialization—total (low > adequate)	*Clostridia*	0.05
socialization—total (low > high)	*Desulfovibrionales*	0.01
socialization—total (adequate > low)	*Enterobacterales*	0.05
	*Veillonellales/Selenomonadales*	0.01
socialization—total (adequate > high)	*Desulfovibrionales*	0.01
using large muscles (high > low)	*Bifidobacteriales*	0.05
using small muscles (adequate > low)	*Clostridiales*	0.01
	*Peptostreptococcales/Tissierellales*	0.05
using small muscles (adequate > high)	*Monoglobales*	0.05
using small muscles (high > low)	*Clostridia*	0.05
	*Clostridiales*	0.01
	*Peptostreptococcales/Tissierellales*	0.05
motor skills—total (adequate > low)	*Monoglobales*	0.05
motor skills—total (high > low)	*Clostridiales*	0.05
motor skills—total (high > adequate)	*Clostridiales*	0.01
Genus		
age (preschoolers > school children)	*Enterorhabdus*	0.05
age (school children > preschoolers)	*Adlercreutzia*	0.05
	*Olsenella*	0.05
	*Lachnospira*	0.05
	*Subdoligranulum*	0.05
	*Romboutsia*	0.05
age (school children > adolescents)	*Clostridium sensu stricte*	0.05
	*Lachnospiraceae NK4A136*	0.05
age (adolescents > school children)	*Klebsiella*	0.01
	*Lactobacillus*	0.05
	*Olsenella*	0.001
diagnosis: childhood autism	*Ruminococcus torques*	0.01
	*GCA.900066575*	0.05
diagnosis: other	*Enterorhabdus*	0.05
sex: female	*Eubacterium ruminantium*	0.05
	*Coprobacillus*	0.05
	*Odoribacter*	0.05
	*UCG010*	0.05
sex: male	*Ruminococcus gravus*	0.05
	*Lachnospiraceae FCS020*	0.05
	*Lautropia*	0.05
	*Peptococcus*	0.05
	*Marvinbryantia*	0.05
vaginal delivery	*Faecalitalea*	0.05
Caesarian’s section	*Eubacterium eligens*	0.05
	*F0332*	0.05
	*Sellimonas*	0.05
	*Clostridium sensu stricte*	0.05
high protein diet	*Eubacterium brachy*	0.05
low-calorie diet	*Weissella*	0.05
high-calorie diet	*Anaerostipes*	0.05
	*Blautia*	0.05
	*Slackia*	0.05
	*Staphylococcus*	0.05
	*TM7x*	0.05
no food selectivity	*Clostridium sensu stricte*	0.05
	*Eubacterium coprostanoligenes*	0.05
	*Barnesiella*	0.05
	*Coprococcus*	0.05
	*DTU089*	0.01
	*Lachnospiraceae NK4A136*	0.05
	*Turicibacter*	0.05
FGID	*Enterobacter*	0.05
	*F0332*	0.01
	*Holdemania*	0.05
	*Oscillibacter*	0.05
	*Clostridium innocuum*	0.05
	*Staphylococcus*	0.05
no FGID	*Lachnospiraceae UCG004*	0.05
bloating	*Eubacterium coprostanoligenes*	0.05
no bloating	*Dielma*	0.05
	*Oxalobacter*	0.05
	*Eubacterium eligens*	0.05
diarrhoea	*Enterobacter*	0.05
	*Family XII UCG001*	0.05
	*Oscillibacter*	0.05
	*Papillibacter*	0.05
	*F0332*	0.05
no diarrhoea	*Oxalobacter*	0.05
constipation	*Clostridium innocuum*	0.05
	*Oscillibacter*	0.05
	*Staphylococcus*	0.01
	*Lachnospira*	0.05
no constipation	*Agathobacter*	0.05
	*Lactococcus*	0.05
communication—expressive skills (adequate > low)	*Clostridium sensu stricto*	0.001
	*Dolosigranulum*	0.05
	*Faecalibacterium*	0.05
	*Lachnospiraceae UCG003*	0.05
	*Romboutsia*	0.05
communication—expressive skills (high > low)	*CAG352*	0.05
	*Eubacterium brachy*	0.05
	*Eubacterium coprostanoligenes*	0.05
	*Dialister*	0.01
	*Enterococcus*	0.05
	*Flavonifractor*	0.05
	*Incertae sedis*	0.05
	*Intestinibacter*	0.05
	*Romboutsia*	0.05
	*Ruminiclostridium*	0.05
	*Veillonella*	0.05
communication—receptive skills (low > adequate)	*Erysipelotrichaceae UCG003*	0.05
communication—receptive skills (adequate > low)	*GCA.900066575*	0.01
	*Tyzzerella*	0.05
communication—receptive skills (adequate > high)	*Faecalitalea*	0.05
communication—receptive skills (high > low)	*CAG352*	0.05
	*Eisenbergiella*	0.05
	*Enorma*	0.05
	*Enterococcus*	0.01
	*Flavonifractor*	0.01
	*GCA.900066575*	0.01
	*Hungatella*	0.05
	*Intestinibacter*	0.05
	*Ruminiclostridium*	0.01
	*Tyzzerella*	0.05
communication—receptive skills (high > adequate)	*Dialister*	0.05
	*Insertae sedis*	0.05
communication—writing skills (low > adequate)	*Blautia*	0.05
communication—writing skills (low > high)	*Ruminococcus gauvreauli*	0.05
	*Collinsella*	0.05
	*Dielma*	0.05
communication—writing skills (adequate > low)	*Catenisphaera*	0.05
	*Lachnospiraceae UCG.003*	0.05
	*Pseudochrobactrum*	0.001
	*Pseudomonas*	0.001
	*Tyzzerella*	0.01
communication—writing skills (high > low)	*Hungatella*	0.05
communication—total (low > adequate)	*Actinomyces*	0.05
	*Gemella*	0.05
	*Granullicatella*	0.05
	*Streptococcus*	0.05
communication—total (low > high)	*Defluviitaleaceae UCG.011*	0.05
	*GCA.900066575*	0.05
	*Pseudochrobactrum*	0.05
	*Pseudomonas*	0.05
	*Pseudopropionibacterium*	0.05
	*Ruminiclostridium*	0.05
communication—total (adequate > low)	*Dielma*	0.05
	*Lachnospiraceae NK4A136*	0.05
	*Oscillibacter*	0.05
	*Paraprevotella*	0.05
	*Phascolarctobacterium*	0.05
	*Ruminiclostridium*	0.05
communication—total (adequate > high)	*Phascolarctobacterium*	0.05
communication—total (high > low)	*Anaerococcus*	0.05
	*CAG.352*	0.01
	*Eisenbergiella*	0.01
	*Enorma*	0.05
	*Enterococcus*	0.05
	*Flavonifractor*	0.05
	*Gemella*	0.05
	*Hungatella*	0.05
	*Tyzzerella*	0.05
communication—total (high > adequate)	*Granullicatella*	0.05
	*Streptococcus*	0.05
daily living skill personal (low > high)	*Collinsella*	0.05
daily living skill personal (adequate > low)	*Clostidium sensu stricto*	0.05
	*Veillonella*	0.01
	*Dolosigranulum*	0.05
	*Lachnospiraceae FCS020*	0.05
	*Sarcina*	0.05
daily living skill personal (high > low)	*CAG.352*	0.05
	*Eubacterium brachy*	0.05
	*Eubacterium coprostanoligenes*	0.05
	*Dialister*	0.05
	*Flavonifractor*	0.05
	*Incertae sedis*	0.05
	*Intestinibacter*	0.05
	*Romboutsia*	0.05
	*Veillonella*	0.05
daily living skill personal (high > adequate)	*Anaerococcus*	0.05
	*CAG.352*	0.05
	*Colidextribacter*	0.05
	*GCA.900066575*	0.05
daily living skill domestic (low > adequate)	*Ruminiclostridium*	0.05
daily living skill domestic (low > high)	*Anaerostipes*	0.05
	*Butyricicoccus*	0.05
	*Lachnospiraceae UCG.003*	0.05
	*Oxalobacter*	0.05
	*Pseudochrobacter*	0.05
	*Pseudomonas*	0.05
daily living skill domestic (adequate > low)	*Butyricimonas*	0.05
	*Clostridium sensu stricto*	0.01
	*Coprococcus*	0.05
	*DTU089*	0.05
	*Intestinibacter*	0.05
	*Veillonella*	0.05
daily living skill domestic (high > low)	*Dialister*	0.05
	*Enterococcus*	0.05
	*Escherichia/Shigella*	0.05
	*Hungatella*	0.05
	*Incertae sedis*	0.05
	*Intestinibacter*	0.05
	*Papillibacter*	0.05
	*Tyzzerella*	0.01
daily living skills—community skills (low > adequate)	*Ruminococcus gnavus*	0.05
daily living skills—community skills (low > high)	*Granullicatella*	0.05
	*Parvimonas*	0.05
daily living skill community (adequate > low)	*Barnesiella*	0.05
	*Eubacterium siraeum*	0.05
	*Coprococcus*	0.05
	*Dialister*	0.01
	*Lachnospiraceae FCS020*	0.01
	*NK4A214*	0.05
	*Odoribacter*	0.05
	*Oscillibacter*	0.01
	*Parabacteroides*	0.05
	*Ruminiclostridium*	0.01
	*Sarcina*	0.05
	*Turicibacter*	0.05
	*UCG.003*	0.05
daily living skill community (adequate > high)	*Agathobacter*	0.05
	*Allistipes*	0.01
	*Barnesiella*	0.05
	*Ruminococcus gauvreaui*	0.05
	*Haemophilus*	0.05
	*Parabacteroides*	0.05
	*UCG.003*	0.05
daily living skills—community skills (high > low)	*Escherichia/Shigella*	0.05
	*Hungatella*	0.05
	*Incertae sedis*	0.05
	*Senegalimassilia*	0.05
	*Tyzzerella*	0.05
daily living skills—community skills (high > adequate)	*Ruminococcus gnavus*	0.05
daily living skills—total (low > high)	*Anaerostipes*	0.05
	*Butyricicoccus*	0.05
	*Lachnospiraceae UCG.003*	0.05
	*Oxalobacter*	0.05
	*Pseudochrobactrum*	0.05
	*Pseudomonas*	0.05
daily living skill total (adequate > low)	*Butyricimonas*	0.05
	*Clostidium sensu stricto*	0.05
	*Oscillibacter*	0.05
	*Ruminiclostridium*	0.05
	*Staphylococcus*	0.05
daily living skill total (adequate > high)	*Anaerostipes*	0.05
daily living skills—total (high > low)	*Dialister*	0.05
	*Enterococcus*	0.05
	*Escherichia/Shigella*	0.05
	*Flavonifractor*	0.05
	*Hungatella*	0.05
	*Papillibacter*	0.05
	*Tyzzerella*	0.01
socialization—interpersonal skills (low > adequate)	*Ruminiclostridium*	0.05
socialization—interpersonal skills (low > high)	*Anaerostipes*	0.05
	*Barnesiella*	0.05
	*Butyricicoccus*	0.05
	*Collinsella*	0.05
socialization—interpersonal skills (adequate > low)	*Ruminococcus torques*	0.05
	*Coprococcus*	0.05
	*Dialister*	0.05
	*Lachnospiraceae FCS020*	0.05
	*Odoribacter*	0.05
	*Subdoligranulum*	0.01
socialization—interpersonal skills (adequate > high)	*Barnesiella*	0.05
	*Butyricicoccus*	0.05
	*Lachnospiraceae NK4A136*	0.05
	*Monoglobus*	0.05
socialization—interpersonal skills (high > low)	*Ruminococcus gnavus*	0.05
	*F0032*	0.05
	*Lachnospiraceae UCG.003*	0.05
	*Oxalobacter*	0.05
	*Veillonella*	0.01
socialization—interpersonal skills (high > adequate)	*Clostidium innocuum*	0.05
	*Ruminococcus gnavus*	0.05
socialization—play and leisure skills (adequate > low)	*Barnesiella*	0.05
	*Odoribacter*	0.05
	*Ruminiclostridium*	0.05
	*UCG.003*	0.05
	*Veillonella*	0.05
socialization—play and leisure skills (high > low)	*CAG.352*	0.01
	*Dialister*	0.05
	*Enorma*	0.05
	*Pseudochrobactrum*	0.01
	*Pseudomonas*	0.01
socialization—coping skills (adequate > low)	*Actinomyces*	0.05
	*Eubacterium brachy*	0.01
	*F0332*	0.05
	*Hungatella*	0.05
	*Ruminococcus torques*	0.05
	*TM7x*	0.01
	*Veillonella*	0.05
	*Weissella*	0.01
socialization—coping skills (high > low)	*Eubacterium brachy*	0.01
	*Defluviitaleaceae UCG.011*	0.001
	*Dialister*	0.05
	*Dorea*	0.05
	*Enorma*	0.001
	*Enterobacter*	0.05
	*F0332*	0.001
	*Flavonifractor*	0.05
	*Oscillibacter*	0.05
	*Parasutterella*	0.01
	*Pseudopropionibacterium*	0.001
	*Ruminiclostridium*	0.05
	*TM7x*	0.01
	*Varibaculum*	0.05
socialization—coping skills (high > adequate)	*Parasutterella*	0.05
socialization—total (low > high)	*Enorma*	0.05
socialization—total (adequate > low)	*Clostridium sensu stricto*	0.05
	*Dialister*	0.05
	*Enterobacter*	0.05
	*Enterococcus*	0.05
	*Lachnospira*	0.05
	*Subdoligranulum*	0.05
socialization—total (high > low)	*Bilophila*	0.01
	*F0332*	0.05
	*Oscillibacter*	0.05
	*Parasutterella*	0.05
socialization—total (high > adequate)	*Bilophila*	0.01
	*Olsenella*	0.01
using large muscles (adequate > low)	*Romboutsia*	0.05
using large muscles (high > low)	*Bifidobacterium*	0.05
using large muscles (high > adequate)	*Flavonifractor*	0.05
	*Staphylococcus*	0.05
	*TM7x*	0.05
using small muscles (adequate > low)	*Clostridium sensu stricto*	0.05
	*Coprococcus*	0.05
	*Dolosigranulum*	0.05
	*Lachnospiraceae FCS020*	0.05
	*Romboutsia*	0.05
	*Veillonella*	0.01
using small muscles (adequate > high)	*Monoglobus*	0.05
using small muscles (high > low)	*Butyricimonas*	0.05
	*Clostridium sensu stricto*	0.05
	*Eubacterium coprostanoligenes*	0.05
	*Ruminococcus torques*	0.05
	*Incertae sedis*	0.05
	*Intestinibacter*	0.05
	*Parasutterella*	0.05
	*Romboutsia*	0.05
	*Ruminiclostridium*	0.05
	*Sarcina*	0.01
	*UCG.005*	0.05
	*Veillonella*	0.05
using small muscles (high > adequate)	*Butyricimonas*	0.05
	*Sarcina*	0.05
motor skills—total (adequate > low)	*Eubacterium siraeum*	0.01
	*Eubacterium ventriosum*	0.05
	*Ruminococcus gauvreauii*	0.01
	*Ruminococcus torques*	0.01
	*Coprococcus*	0.05
	*Frisingicoccus*	0.05
	*Lachnospiraceae FCS020*	0.05
	*Monoglobus*	0.05
motor skills—total (high > low)	*Clostridium sensu stricto*	0.05
	*Eubacterium coprostanoligenes*	0.05
	*Intestinibacter*	0.05
	*Lachnospiraceae FCS020*	0.05
motor skills—total (high > adequate)	*Clostridium sensu stricto*	0.01
	*Intestinibacter*	0.01
Species		
age (adolescents > school children)	*Clostridum perfingens*	0.01
	*Lactobacillus paracasei*	0.01
	*Lactobacillus sakei*	0.05
	*Pediococcus pentosaceus*	0.01
diagnosis: childhood autism	*Streptococcus mutans*	0.05
sex: female	*Lactobacillus fermentum*	0.01
	*Streptococcus sobrinus*	0.01
high-protein diet	*Lactobacillus curvatus*	0.05
FGID	*Leuconostoc mesenteroides*	0.05
diarrhoea	*Leuconostoc lactis*	0.05
	*Leuconostoc citreum*	0.05
no diarrhoea	*Enterobacter cloacae*	0.05
	*Lactobacillus oligofermentans*	0.05
	*Lactobacillus brevis*	0.05
bloating	*Eubacterium hallii*	0.05
no bloating	*Lactobacillus delbrueckii*	0.05
	*Pediococcus pentosaceus*	0.05
constipation	*Eubacterium hallii*	0.05
no constipation	*Lactococcus lactis*	0.05
communication—expressive skills (high > low)	*Lactococcus garvieae*	0.05
communication—receptive skills (adequate > low)	*Leuconostoc citreum*	0.05
	*Leuconostoc lactis*	0.05
communication—receptive skills (high > low)	*Lactococcus garvieae*	0.01
communication—writing skills (low > high)	*Clostridium tertium*	0.05
communication—writing skills (adequate > low)	*Enterococcus casseliflavus*	0.001
	*Enterococcus faecalis*	0.001
	*Lactobacillus fermentum*	0.05
	*Pseudomonas* spp.	0.001
	*Streptococcus sobrinus*	0.05
communication—writing skills (adequate > high)	*Bifidobacterium bifidum*	0.05
communication—total (low > high)	*Clostridium tertium*	0.05
	*Enterococcus casseliflavus*	0.05
	*Enterococcus faecalis*	0.05
	*Haemophilus pittmaniae*	0.05
	*Pseudomonas* spp.	0.05
communication—total (high > low)	*Bifidobacterium animalis*	0.05
	*Lactococcus garvieae*	0.01
communication—total (high > adequate)	*Streptococcus salivarius*	0.01
daily living skill personal (adequate > low)	*Odoribacter splanchnicus*	0.05
daily living skill personal (high > low)	*Lactococcus garvieae*	0.05
daily living skill domestic (low > adequate)	*Staphylococcus epidermidis*	0.01
	*Streptococcus gallolyticus*	0.05
	*Streptococcus sobrinus*	0.05
daily living skill domestic (low > high)	*Clostridium tertium*	0.05
	*Enterococcus casseliflavus*	0.05
	*Enterococcus faecalis*	0.05
	*Lactococcus garvieae*	0.05
	*Pseudomonas* spp.	0.05
daily living skill domestic (adequate > low)	*Lactobacillus fermentum*	0.05
	*Odoribacter splanchnicus*	0.01
daily living skill community (adequate > low)	*Lactobacillus fermentum*	0.01
	*Lactobacillus salivarius*	0.05
	*Odoribacter splanchnicus*	0.01
	*Streptococcus sobrinus*	0.01
daily living skill total (low > high)	*Clostridium tertium*	0.05
	*Enterococcus casseliflavus*	0.05
	*Enterococcus faecalis*	0.05
	*Lactococcus garvieae*	0.05
	*Pseudomonas* spp.	0.05
socialization—interpersonal skills (low > adequate)	*Lactobacillus fermentum*	0.05
	*Streptococcus sobrinus*	0.05
socialization—interpersonal skills (adequate > low)	*Streptococcus mutans*	0.05
socialization—interpersonal skills (high > low)	*Clostridium tertium*	0.05
	*Lactococcus garvieae*	0.05
	*Staphylococcus haemolyticus*	0.05
socialization—play and leisure skills (adequate > low)	*Lactobacillus fermentum*	0.05
	*Lactococcus garvieae*	0.05
	*Odoribacter splanchnicus*	0.05
	*Streptococcus sobrinus*	0.05
socialization—play and leisure skills (high > low)	*Enterococcus casseliflavus*	0.01
	*Enterococcus faecalis*	0.01
	*Pseudomonas* spp.	0.01
socialization—coping skills (adequate > low)	*Leuconostoc citreum*	0.01
	*Leuconostoc lactis*	0.01
socialization—coping skills (high > low)	*Haemophilus pittmaniae*	0.001
	*Lactococcus garvieae*	0.001
	*Leuconostoc citreum*	0.001
	*Streptococcus salivarius*	0.05
socialization—total (low > adequate)	*Lactococcus garvieae*	0.05
using large muscles (adequate > low)	*Streptococcus mutans*	0.05
using small muscles (high > low)	*Bifidobacterium bifidum*	0.01
	*Lactobacillus salivarius*	0.05
	*Lactococcus garvieae*	0.05
	*Streptococcus anginosus*	0.05
motor skills—total (adequate > low)	*Eubacterium hallii*	0.05
motor skills—total (high > adequate)	*Bifidobacterium bifidum*	0.05

FGID—functional gastrointestinal disorders.

**Table 3:** Table of correlations among various features and gut microbiota composition.

Feature	Bacteria	Correlation (r)
Order		
age	*Micrococcales*	−0.47
fat saturated (mean)/fat total (mean)	*Peptostreptococcales/Tissierellales*	−0.46
complex carbohydrates (mean)	*Clostridiales*	0.5
	*Peptostreptococcales/Tissierellales*	0.47
daily living skills—personal skills (v-score)	*Peptostreptococcales/Tissierellales*	0.5
using small muscles (v-score)	*Clostridiales*	0.53
	*Peptostreptococcales/Tissierellales*	0.57
Genus		
age	*Rothia*	−0.47
fat saturated (mean)/fat total (mean)	*Eubacterium coprostanoligenes*	−0.46
	*Eubacterium hallii*	−0.59
	*Dorea*	−0.6
	*Leucostonoc*	0.49
fat unsaturated (mean)/fat total (mean)	*Dielma*	0.55
fat (mean)/weight (kg)	*Dielma*	0.47
simple carbohydrates (mean)/carbohydrates total (mean)	*Epulopiscium*	0.53
	*Flavonifractor*	−0.5
	*Weissella*	0.51
complex carbohydrates (mean)	*Romboutsia*	0.52
kcal from carbohydrates/total carbohydrates (mean)	*Epulopiscium*	0.5
	*Klebsiella*	0.66
	*Weissella*	0.51
kcal from carbohydrates/total kcal	*Epulopiscium*	0.5
	*Klebsiella*	0.66
protein (mean)/weight (kg)	*Dielma*	0.52
communication—receptive skills (v-score)	*Flavonifractor*	0.49
communication—writing skills (v-score)	*Anaerostipes*	−0.51
daily living skills—personal skills (v-score)	*Romboutsia*	0.5
daily living skills—domestic skills (v-score)	*Intestinibacter*	0.5
socialization—coping skills (v-score)	*F0032*	0.5
using small muscles (v-score)	*Romboutsia*	0.55
Species		
fat saturated (mean)/fat total (mean)	*Enterobacter cloacae*	0.68
	*Leuconostoc citreum*	0.53
	*Leuconostoc lactis*	0.56
	*Staphylococcus epidermidis*	0.46
	*Streptococcus gallolyticus*	0.66
kcal from carbohydrates/total carbohydrates (mean)	*Clostridium perfingens*	0.62

Statistical methods applied to the data in Table 3: Pearson’s correlation coefficient was used for all correlations, with the exception of those involving mean complex carbohydrates and age, where Spearman’s rank correlation coefficient was employed.

## Data Availability

The raw data supporting the conclusions of this article will be made available by the authors on request due to privacy of the patients, including regional GDPR regulations.

## Abbreviations

The following abbreviations are used in this manuscript:

AD	Alzheimer’s disease
ASD	Autism Spectrum Disorder
ChBD	carbohydrate-based diet
CNS	central nervous system
FGID	Functional Gastrointestinal Disorder
FS	food selectivity
GABA	γ-aminobutyric acid
HCD	high-calorie diet
IBS	irritable bowel syndrome
ICD-10	International Statistical Classification of Diseases and Related Health Problems—10th Revision
LCD	low-calorie diet
MDD	major depressive disorder
MS	multiple sclerosis
MSA	multiple system atrophy
PBD	protein-based diet
PD	Parkinson’s disease
RS	Rett syndrome
SCFA	short-chain fatty acid
SMA	spinal muscular atrophy
VABS	Vineland Adaptive Behavior Scales

## References

[1] World Health Organization Autism. https://www.who.int/news-room/fact-sheets/detail/autism-spectrum-disorders.

[2] Zeidan J., Fombonne E., Scorah J., Ibrahim A., Durkin M.S., Saxena S., Yusuf A., Shih A., Elsabbagh M. (2022). Global prevalence of autism: A systematic review update. Autism Res..

[3] ICD-10 Version: 2019. Pervasive Developmental Disorders. https://icd.who.int/browse10/2019/en#/F84.

[4] Tye C., Runicles A.K., Whitehouse A.J.O., Alvares G.A. (2019). Characterizing the Interplay Between Autism Spectrum Disorder and Comorbid Medical Conditions: An Integrative Review. Front. Psychiatry.

[5] Krajmalnik-Brown R., Lozupone C., Kang D.W., Adams J.B. (2015). Gut Bacteria in Children with Autism Spectrum Disorders: Challenges and Promise of Studying How a Complex Community Influences a Complex Disease. Microb. Ecol. Health Dis..

[6] Arnold L.E., Luna R.A., Williams K., Chan J., Parker R.A., Wu Q., Hollway J.A., Jeffs A., Lu F., Hayes C. (2019). Probiotics for Gastrointestinal Symptoms and Quality of Life in Autism: A Placebo-Controlled Pilot Trial. J. Child. Adolesc. Psychopharmacol..

[7] Lewandowska-Pietruszka Z., Figlerowicz M., Mazur-Melewska K. (2022). The History of the Intestinal Microbiota and the Gut-Brain Axis. Pathogens.

[8] Lewandowska-Pietruszka Z., Figlerowicz M., Mazur-Melewska K. (2023). Microbiota in Autism Spectrum Disorder: A Systematic Review. Int. J. Mol. Sci..

[9] West K.A., Yin X., Rutherford E.M., Wee B., Choi J., Chrisman B.S., Dunlap K.L., Hannibal R.L., Hartono W., Lin M. (2022). Multi-angle meta-analysis of the gut microbiome in Autism Spectrum Disorder: A step toward understanding patient subgroups. Sci. Rep..

[10] Wu T., Wang H., Lu W., Zhai Q., Zhang Q., Yuan W., Gu Z., Zhao J., Zhang H., Chen W. (2020). Potential of gut microbiome for detection of autism spectrum disorder. Microb. Pathog..

[11] Tomova A., Husarova V., Lakatosova S., Bakos J., Vlkova B., Babinska K., Ostatnikova D. (2015). Gastrointestinal microbiota in children with autism in Slovakia. Physiol. Behav..

[12] Son J.S., Zheng L.J., Rowehl L.M., Tian X., Zhang Y., Zhu W., Litcher-Kelly L., Gadow K.D., Gathungu G., Robertson C.E. (2015). Comparison of Fecal Microbiota in Children with Autism Spectrum Disorders and Neurotypical Siblings in the Simons Simplex Collection. PLoS ONE.

[13] Grimaldi R., Gibson G.R., Vulevic J., Giallourou N., Castro-Mejía J.L., Hansen L.H., Leigh Gibson E., Nielsen D.S., Costabile A. (2018). A prebiotic intervention study in children with autism spectrum disorders (ASDs). Microbiome.

[14] Tomova A., Soltys K., Kemenyova P., Karhanek M., Babinska K. (2020). The Influence of Food Intake Specificity in Children with Autism on Gut Microbiota. Int. J. Mol. Sci..

[15] Lewandowska-Pietruszka Z., Figlerowicz M., Mazur-Melewska K. (2025). Oral Microbiota Composition and Its Association with Gastrointestinal and Developmental Abnormalities in Children with Autism Spectrum Disorder. Microorganisms.

[16] Paudel D., Uehara O., Giri S., Yoshida K., Morikawa T., Kitagawa T., Matsuoka H., Miura H., Toyofuku A., Kuramitsu Y. (2022). Effect of psychological stress on the oral-gut microbiota and the potential oral-gut-brain axis. Jpn. Dent. Sci. Rev..

[17] Carabotti M., Scirocco A., Maselli M.A., Severi C. (2015). The gut-brain axis: Interactions between enteric microbiota, central and enteric nervous systems. Ann. Gastroenterol..

[18] Klepacz N., Rabęda A., Grzesik K., Pilarczyk K., Adamska H., Kaus M., Nowak W.E., Sawczuk H., Cudziło Z., Malicka M. (2025). The microbiome-mind connection: Exploring gut health’s impact on depression. The microbiome-mind connection: Exploring gut health’s impact on depression. J. Med. Sci..

[19] Lewandowska Z., Mazur-Melewska K., Figlerowicz M. (2020). Cortisol in individuals with autism spectrum disorders—Review. Neurol. Dziecięca.

[20] Dao M.C., Subar A.F., Warthon-Medina M., Cade J.E., Burrows T., Golley R.K., Forouhi N.G., Pearce M., Holmes B.A. (2018). Dietary assessment toolkits: An overview. Public Health Nutr..

[21] Pearce M., Powell R., Imamura F., De Lucia Rolfe E., Brage S., Forouhi N. Estimated Food Diaries. Measurement Toolkit. https://www.measurement-toolkit.org/diet/subjective-methods/estimated-food-diaries.

[22] Sawicka-Gutaj N., Gruszczyński D., Guzik P., Mostowska A., Walkowiak J. (2022). Publication ethics of human studies in the light of the Declaration of Helsinki—A mini-review. J. Med. Sci..

[23] Shao Y., Forster S.C., Tsaliki E., Vervier K., Strang A., Simpson N., Kumar N., Stares M.D., Rodger A., Brocklehurst P. (2019). Stunted microbiota and opportunistic pathogen colonisation in caesarean section birth. Nature.

[24] Nakhal M.M., Yassin L.K., Alyaqoubi R., Saeed S., Alderei A., Alhammadi A., Alshehhi M., Almehairbi A., Al Houqani S., BaniYas S. (2024). The Microbiota-Gut-Brain Axis and Neurological Disorders: A Comprehensive Review. Life.

[25] Winiarska-Mieczan A., Tomaszewska E., Donaldson J., Jachimowicz K. (2022). The Role of Nutritional Factors in the Modulation of the Composition of the Gut Microbiota in People with Autoimmune Diabetes. Nutrients.

[26] Ioannou P., Kampanieri E., Koukias S., Baliou S., Tsantes A.G., Kofteridis D. (2025). Kytococcus Species Infections in Humans-A Narrative Review. Microorganisms.

[27] Marples R.R., Richardson J.F. (1980). Micrococcus in the blood. J. Med. Microbiol..

[28] Fatahi-Bafghi M. (2021). Characterization of the *Rothia* spp. and their role in human clinical infections. Infect. Genet. Evol..

[29] Radjabzadeh D., Bosch J.A., Uitterlinden A.G., Zwinderman A.H., Ikram M.A., van Meurs J.B.J., Luik A.I., Nieuwdorp M., Lok A., van Duijn C.M. (2022). Gut microbiome-wide association study of depressive symptoms. Nat. Commun..

[30] Barandouzi Z.A., Starkweather A.R., Henderson W.A., Gyamfi A., Cong X.S. (2020). Altered Composition of Gut Microbiota in Depression: A Systematic Review. Front. Psychiatry.

[31] Li P., Wang S., Li J., Xiao Z., Zhu H., Sheng D., Liu W., Xiao B., Zhou L. (2025). Appraising the Effects of Gut Microbiota on Insomnia Risk Through Genetic Causal Analysis. Am. J. Med. Genet. B Neuropsychiatr. Genet..

[32] Gao M., Wang J., Liu P., Tu H., Zhang R., Zhang Y., Sun N., Zhang K. (2023). Gut microbiota composition in depressive disorder: A systematic review, meta-analysis, and meta-regression. Transl. Psychiatry.

[33] Oñate F.P., Chamignon C., Burz S.D., Lapaque N., Monnoye M., Philippe C., Bredel M., Chêne L., Farin W., Paillarse J.M. (2023). *Adlercreutzia equolifaciens* is an Anti-Inflammatory Commensal Bacterium with Decreased Abundance in Gut Microbiota of Patients with Metabolic Liver Disease. Int. J. Mol. Sci..

[34] Martinez-Guryn K., Leone V., Chang E.B. (2019). Regional Diversity of the Gastrointestinal Microbiome. Cell Host Microbe.

[35] Winpenny E.M., van Sluijs E.M.F., White M., Klepp K.I., Wold B., Lien N. (2018). Changes in diet through adolescence and early adulthood: Longitudinal trajectories and association with key life transitions. Int. J. Behav. Nutr. Phys. Act..

[36] Jiang S., Cai L., Lv L., Li L. (2021). Pediococcus pentosaceus, a future additive or probiotic candidate. Microb. Cell Fact..

[37] Iseppi R., Messi P., Camellini S., Sabia C. (2019). Bacteriocin activity of *Lactobacillus brevis* and *Lactobacillus paracasei* ssp. paracasei. J. Med. Microbiol..

[38] Zou X., Pan L., Xu M., Wang X., Wang Q., Han Y. (2023). Probiotic potential of *Lactobacillus sakei* L-7 in regulating gut microbiota and metabolism. Microbiol. Res..

[39] Raimondi S., Musmeci E., Candeliere F., Amaretti A., Rossi M. (2021). Identification of mucin degraders of the human gut microbiota. Sci. Rep..

[40] Guo L., Lan Q., Zhou M., Liu F. (2025). From gut to kidney: Microbiota modulates stone risk through inflammation–a mediated Mendelian randomization study. Mamm. Genome.

[41] Shen Y., Li C., Zhang X., Wang Y., Zhang H., Yu Z., Gui B., Hu R., Li Q., Gao A. (2024). Gut microbiota linked to hydrocephalus through inflammatory factors: A Mendelian randomization study. Front. Immunol..

[42] Ma J., Zhu Z., Yishajiang Y., Alarjani K.M., Hong L., Luo L. (2023). Role of gut microbiota and inflammatory factors in acute respiratory distress syndrome: A Mendelian randomization analysis. Front. Microbiol..

[43] Wen J., He J.Q. (2023). The Causal Impact of the Gut Microbiota on Respiratory Tuberculosis Susceptibility. Infect. Dis. Ther..

[44] Crost E.H., Coletto E., Bell A., Juge N. (2023). *Ruminococcus gnavus*: Friend or foe for human health. FEMS Microbiol Rev..

[45] Genseke S., Berisha M., Teerstegen A., Meyer B., Kaasch A.J., Färber J., Schalk E., Zautner A.E., Esser T., Kahlfuß S. (2025). *Lautropia mirabilis* sepsis in immunodeficiency: First report and genomic features. Infection.

[46] Tap J., Derrien M., Törnblom H., Brazeilles R., Cools-Portier S., Doré J., Störsrud S., Le Nevé B., Öhman L., Simrén M. (2017). Identification of an Intestinal Microbiota Signature Associated with Severity of Irritable Bowel Syndrome. Gastroenterology.

[47] Li H., Liu C., Huang S., Wang X., Cao M., Gu T., Ou X., Pan S., Lin Z., Wang X. (2023). Multi-omics analyses demonstrate the modulating role of gut microbiota on the associations of unbalanced dietary intake with gastrointestinal symptoms in children with autism spectrum disorder. Gut Microbes.

[48] Verhaar B.J.H., Hendriksen H.M.A., de Leeuw F.A., Doorduijn A.S., van Leeuwenstijn M., Teunissen C.E., Barkhof F., Scheltens P., Kraaij R., van Duijn C.M. (2022). Gut Microbiota Composition Is Related to AD Pathology. Front. Immunol..

[49] Yang Y., Wen C., Zheng S., Song F., Liu Y., Yao X., Tang Y., Feng X., Chen J., Yang F. (2023). *Lactobacillus fermentum* Alleviates the Colorectal Inflammation Induced by Low-Dose Sub-Chronic Microcystin-LR Exposure. Toxins.

[50] Lee J., d’Aigle J., Atadja L., Quaicoe V., Honarpisheh P., Ganesh B.P., Hassan A., Graf J., Petrosino J., Putluri N. (2020). Gut Microbiota-Derived Short-Chain Fatty Acids Promote Poststroke Recovery in Aged Mice. Circ. Res..

[51] Moriki D., León E.D., García-Gamero G., Jiménez-Hernández N., Artacho A., Pons X., Koumpagioti D., Dinopoulos A., Papaevangelou V., Priftis K.N. (2024). Specific Gut Microbiome Signatures in Children with Cow’s Milk Allergy. Nutrients.

[52] Keskitalo A., Munukka E., Toivonen R., Hollmén M., Kainulainen H., Huovinen P., Jalkanen S., Pekkala S. (2018). Enterobacter cloacae administration induces hepatic damage and subcutaneous fat accumulation in high-fat diet fed mice. PLoS ONE.

[53] Yan H., Fei N., Wu G., Zhang C., Zhao L., Zhang M. (2016). Regulated Inflammation and Lipid Metabolism in Colon mRNA Expressions of Obese Germfree Mice Responding to Enterobacter cloacae B29 Combined with the High Fat Diet. Front. Microbiol..

[54] Bai X.B., Xu S., Zhou L.J., Meng X.Q., Li Y.L., Chen Y.L., Jiang Y.H., Lin W.Z., Chen B.Y., Du L.J. (2023). Oral pathogens exacerbate Parkinson’s disease by promoting Th1 cell infiltration in mice. Microbiome.

[55] Nishiwaki H., Ueyama J., Kashihara K., Ito M., Hamaguchi T., Maeda T., Tsuboi Y., Katsuno M., Hirayama M., Ohno K. (2022). Gut microbiota in dementia with Lewy bodies. NPJ Park. Dis..

[56] Wang Q., Song Y.X., Wu X.D., Luo Y.G., Miao R., Yu X.M., Guo X., Wu D.Z., Bao R., Mi W.D. (2024). Gut microbiota and cognitive performance: A bidirectional two-sample Mendelian randomization. J. Affect. Disord..

[57] Akinkunmi E.O., Adeyemi O.I., Igbeneghu O.A., Olaniyan E.O., Omonisi A.E., Lamikanra A. (2014). The pathogenicity of Staphylococcus epidermidis on the intestinal organs of rats and mice: An experimental investigation. BMC Gastroenterol..

[58] Zhu Z., Ma X., Wu J., Xiao Z., Wu W., Ding S., Zheng L., Liang X., Luo J., Ding D. (2022). Altered Gut Microbiota and Its Clinical Relevance in Mild Cognitive Impairment and Alzheimer’s Disease: Shanghai Aging Study and Shanghai Memory Study. Nutrients.

[59] Ning J., Huang S.Y., Chen S.D., Zhang Y.R., Huang Y.Y., Yu J.T. (2022). Investigating Casual Associations Among Gut Microbiota, Metabolites, and Neurodegenerative Diseases: A Mendelian Randomization Study. J. Alzheimer’s Dis..

[60] Strati F., Cavalieri D., Albanese D., De Felice C., Donati C., Hayek J., Jousson O., Leoncini S., Pindo M., Renzi D. (2016). Altered gut microbiota in Rett syndrome. Microbiome.

[61] Lee S.H., Han C., Shin C. (2025). IUPHAR review: Microbiota-gut-brain axis and its role in neuropsychiatric disorders. Pharmacol. Res..

[62] Aboushaala K., Chee A.V., Adnan D., Toro S.J., Singh H., Savoia A., Dhillon E.S., Yuh C., Dourdourekas J., Patel I.K. (2024). Gut microbiome dysbiosis is associated with lumbar degenerative spondylolisthesis in symptomatic patients. JOR Spine.

[63] Teixeira C.G., Fusieger A., Milião G.L., Martins E., Drider D., Nero L.A., de Carvalho A.F. (2021). Weissella: An Emerging Bacterium with Promising Health Benefits. Probiotics Antimicrob. Proteins.

[64] Molina-López J., Leiva-García B., Planells E., Planells P. (2021). Food selectivity, nutritional inadequacies, and mealtime behavioral problems in children with autism spectrum disorder compared to neurotypical children. Int. J. Eat. Disord..

[65] Huang H., Cheng S., Yang X., Liu L., Cheng B., Meng P., Pan C., Wen Y., Jia Y., Liu H. (2023). Dissecting the Association between Gut Microbiota and Brain Structure Change Rate: A Two-Sample Bidirectional Mendelian Randomization Study. Nutrients.

[66] Nilholm C., Manoharan L., Roth B., D’Amato M., Ohlsson B. (2022). A starch- and sucrose-reduced dietary intervention in irritable bowel syndrome patients produced a shift in gut microbiota composition along with changes in phylum, genus, and amplicon sequence variant abundances, without affecting the micro-RNA levels. United Eur. Gastroenterol. J..

[67] Marizzoni M., Mirabelli P., Mombelli E., Coppola L., Festari C., Lopizzo N., Luongo D., Mazzelli M., Naviglio D., Blouin J.L. (2023). A peripheral signature of Alzheimer’s disease featuring microbiota-gut-brain axis markers. Alzheimer’s Res. Ther..

[68] Xiang Y., Zhang C., Wang J., Cheng Y., Wang L., Tong Y., Yan D. (2023). Identification of host gene-microbiome associations in colorectal cancer patients using mendelian randomization. J. Transl. Med..

[69] Liang L.D., Li S., Huang M.J., Peng H.X., Lu Z.J., Zhang Z.H., Su L.Y., Sooranna S.R., Liu Y., Huang Z.H. (2024). Causal relationship between gut microbiota and puerperal sepsis: A 2-sample Mendelian randomization study. Front. Microbiol..

[70] Lynch J.B., Gonzalez E.L., Choy K., Faull K.F., Jewell T., Arellano A., Liang J., Yu K.B., Paramo J., Hsiao E.Y. (2023). Gut microbiota Turicibacter strains differentially modify bile acids and host lipids. Nat. Commun..

[71] Li L., Buhman K.K., Hartman P.A., Beitz D.C. (1995). Hypocholesterolemic effect of *Eubacterium coprostanoligenes* ATCC 51222 in rabbits. Lett. Appl. Microbiol..

[72] Telle-Hansen V.H., Gaundal L., Bastani N., Rud I., Byfuglien M.G., Gjøvaag T., Retterstøl K., Holven K.B., Ulven S.M., Myhrstad M.C.W. (2022). Replacing saturated fatty acids with polyunsaturated fatty acids increases the abundance of *Lachnospiraceae* and is associated with reduced total cholesterol levels-a randomized controlled trial in healthy individuals. Lipids Health Dis..

[73] Udayappan S., Manneras-Holm L., Chaplin-Scott A., Belzer C., Herrema H., Dallinga-Thie G.M., Duncan S.H., Stroes E.S.G., Groen A.K., Flint H.J. (2016). Oral treatment with *Eubacterium hallii* improves insulin sensitivity in *db*/*db* mice. NPJ Biofilms Microbiomes.

[74] Bonnechère B., Amin N., van Duijn C. (2022). What Are the Key Gut Microbiota Involved in Neurological Diseases? A Systematic Review. Int. J. Mol. Sci..

[75] Saulnier D.M., Riehle K., Mistretta T.A., Diaz M.A., Mandal D., Raza S., Weidler E.M., Qin X., Coarfa C., Milosavljevic A. (2011). Gastrointestinal microbiome signatures of pediatric patients with irritable bowel syndrome. Gastroenterology.

[76] Cuevas-Sierra A., Milagro F.I., Guruceaga E., Cuervo M., Goni L., García-Granero M., Martinez J.A., Riezu-Boj J.I. (2022). A weight-loss model based on baseline microbiota and genetic scores for selection of dietary treatments in overweight and obese population. Clin. Nutr..

[77] Jang S.E., Min S.W. (2020). Amelioration of colitis in mice by *Leuconostoc lactis* EJ-1 by M1 to M2 macrophage polarization. Microbiol. Immunol..

[78] Muthusamy K., Han H.S., Soundharrajan I., Jung J.S., Valan Arasu M., Choi K.C. (2023). A Novel Strain of Probiotic *Leuconostoc citreum* Inhibits Infection-Causing Bacterial Pathogens. Microorganisms.

[79] Taylor J.C., Gao X., Xu J., Holder M., Petrosino J., Kumar R., Liu W., Höök M., Mackenzie C., Hillhouse A. (2021). A type VII secretion system of *Streptococcus gallolyticus* subsp. *gallolyticus* contributes to gut colonization and the development of colon tumors. PLoS Pathog..

[80] Van Samkar A., Brouwer M.C., Pannekoek Y., van der Ende A., van de Beek D. (2015). *Streptococcus gallolyticus* meningitis in adults: Report of five cases and review of the literature. Clin. Microbiol. Infect..

[81] Xie H., Chen J., Chen Q., Zhao Y., Liu J., Sun J., Hu X. (2023). The Diagnostic Value of Gut Microbiota Analysis for Post-Stroke Sleep Disorders. Diagnostics.

[82] LiYa L., XinSheng Z., Xiang H., Zhao L., Lu L., XiuMing L., Ye L., Jing C., KeMing Z., HongChi W. (2024). A cross-sectional survey study on the correlation analysis of nutritional status and intestinal flora in patients with esophageal cancer. Front. Nutr..

[83] Mao R., Yu Q., Li J. (2023). The causal relationship between gut microbiota and inflammatory dermatoses: A Mendelian randomization study. Front. Immunol..

[84] Shi S., Zhang Q., Sang Y., Ge S., Wang Q., Wang R., He J. (2022). Probiotic Bifidobacterium longum BB68S Improves Cognitive Functions in Healthy Older Adults: A Randomized, Double-Blind, Placebo-Controlled Trial. Nutrients.

[85] Ortega M.A., Álvarez-Mon M.A., García-Montero C., Fraile-Martínez Ó., Monserrat J., Martinez-Rozas L., Rodríguez-Jiménez R., Álvarez-Mon M., Lahera G. (2023). Microbiota-gut-brain axis mechanisms in the complex network of bipolar disorders: Potential clinical implications and translational opportunities. Mol. Psychiatry.

[86] Allaart J.G., van Asten A.J., Gröne A. (2013). Predisposing factors and prevention of *Clostridium perfringens*-associated enteritis. Comp. Immunol. Microbiol. Infect. Dis..

[87] Choi M.G., Jung H.K. (2011). Health related quality of life in functional gastrointestinal disorders in Asia. J. Neurogastroenterol. Motil..

[88] Sundas A., Sampath H., Lamtha S.C., Soohinda G., Dutta S. (2024). Psychosocial quality-of-life correlates in functional gastrointestinal disorders. Rev. Gastroenterol. Méx..

[89] Zhuang Z.Q., Shen L.L., Li W.W., Fu X., Zeng F., Gui L., Lü Y., Cai M., Zhu C., Tan Y.L. (2018). Gut Microbiota is Altered in Patients with Alzheimer’s Disease. J. Alzheimers Dis..

[90] Zhou Y., Wang Y., Quan M., Zhao H., Jia J. (2021). Gut Microbiota Changes and Their Correlation with Cognitive and Neuropsychiatric Symptoms in Alzheimer’s Disease. J. Alzheimers Dis..

[91] Petrov V.A., Saltykova I.V., Zhukova I.A., Alifirova V.M., Zhukova N.G., Dorofeeva Y.B., Tyakht A.V., Kovarsky B.A., Alekseev D.G., Kostryukova E.S. (2017). Analysis of Gut Microbiota in Patients with Parkinson’s Disease. Bull. Exp. Biol. Med..

[92] Liu J., Zhang T., Liu X., Wang Q., Zhang H. (2024). Causal effect between gut microbiota and gastroesophageal reflux disease: A bidirectional two-sample Mendelian randomization study. Eur. J. Gastroenterol. Hepatol..

[93] Miyamoto J., Shimizu H., Hisa K., Matsuzaki C., Inuki S., Ando Y., Nishida A., Izumi A., Yamano M., Ushiroda C. (2023). Host metabolic benefits of prebiotic exopolysaccharides produced by *Leuconostoc mesenteroides*. Gut Microbes.

[94] Peng X., Yi X., Deng N., Liu J., Tan Z., Cai Y. (2023). Zhishi Daozhi decoction alleviates constipation induced by a high-fat and high-protein diet via regulating intestinal mucosal microbiota and oxidative stress. Front. Microbiol..

[95] Barnett D., Thijs C., Mommers M., Endika M., Klostermann C., Schols H., Smidt H., Nauta A., Arts I., Penders J. (2025). Why do babies cry? Exploring the role of the gut microbiota in infantile colic, constipation, and cramps in the KOALA birth cohort study. Gut Microbes.

[96] Bhattacharjee D., Flores C., Woelfel-Monsivais C., Seekatz A.M. (2023). Diversity and Prevalence of *Clostridium innocuum* in the Human Gut Microbiota. mSphere.

[97] Al-Fakhrany O.M., Elekhnawy E. (2024). Next-generation probiotics: The upcoming biotherapeutics. Mol. Biol. Rep..

[98] Zou B., Liu S., Dong C., Shen H., Lv Y., He J., Li X., Ruan M., Huang Z., Shu S. (2025). Fecal microbiota transplantation restores gut microbiota diversity in children with active Crohn’s disease: A prospective trial. J. Transl. Med..

[99] Gotoh Y., Nanba F., Shioya N., Sugimura H., Suzuki T. (2020). A dose-finding study for a supplement containing *Lactococcus lactis* subsp. *cremoris* FC in healthy adults with mild constipation. Biosci. Microbiota Food Health.

[100] Duncan S.H., Richardson A.J., Kaul P., Holmes R.P., Allison M.J., Stewart C.S. (2002). *Oxalobacter formigenes* and its potential role in human health. Appl. Environ. Microbiol..

[101] Li Z., Lai J., Zhang P., Ding J., Jiang J., Liu C., Huang H., Zhen H., Xi C., Sun Y. (2022). Multi-omics analyses of serum metabolome, gut microbiome and brain function reveal dysregulated microbiota-gut-brain axis in bipolar depression. Mol. Psychiatry.

[102] Zhang C.Y., Jiang S.J., Cao J.J., Xu Y., Wang X.Y., Li R., Miao Z.W. (2024). Investigating the causal relationship between gut microbiota and gastroenteropancreatic neuroendocrine neoplasms: A bidirectional Mendelian randomization study. Front. Microbiol..

[103] Kim W.J., Hyun J.H., Lee N.K., Paik H.D. (2022). Protective Effects of a Novel *Lactobacillus brevis* Strain with Probiotic Characteristics against *Staphylococcus aureus* Lipoteichoic Acid-Induced Intestinal Inflammatory Response. J. Microbiol. Biotechnol..

[104] Acosta-Rodríguez-Bueno C.P., Abreu y Abreu A.T., Guarner F., Guno M.J.V., Pehlivanoğlu E., Perez M. (2020). *Bacillus clausii* for Gastrointestinal Disorders: A Narrative Literature Review. Adv. Ther..

[105] Novau-Ferré N., Papandreou C., Rojo-Marticella M., Canals-Sans J., Bulló M. (2025). Gut microbiome differences in children with Attention Deficit Hyperactivity Disorder and Autism Spectrum Disorder and effects of probiotic supplementation: A randomized controlled trial. Res. Dev. Disabil..

[106] He J., Gong X., Hu B., Lin L., Lin X., Gong W., Zhang B., Cao M., Xu Y., Xia R. (2023). Altered Gut Microbiota and Short-chain Fatty Acids in Chinese Children with Constipated Autism Spectrum Disorder. Sci. Rep..

[107] Kok C.R., Hutkins R. (2018). Yogurt, and other fermented foods as sources of health-promoting bacteria. Nutr Rev..

[108] Olorocisimo J.P., Diaz L.A., Co D.E., Carag H.M., Ibana J.A., Velarde M.C. (2023). *Lactobacillus delbrueckii* reduces anxiety-like behavior in zebrafish through a gut microbiome—Brain crosstalk. Neuropharmacology.

[109] Xu Y., Yu Y., Shen Y., Li Q., Lan J., Wu Y., Zhang R., Cao G., Yang C. (2021). Effects of *Bacillus subtilis* and *Bacillus licheniformis* on growth performance, immunity, short chain fatty acid production, antioxidant capacity, and cecal microflora in broilers. Poult. Sci..

[110] Muhammad F., Fan B., Wang R., Ren J., Jia S., Wang L., Chen Z., Liu X.A. (2022). The Molecular Gut-Brain Axis in Early Brain Development. Int. J. Mol. Sci..

[111] Marangelo C., Vernocchi P., Del Chierico F., Scanu M., Marsiglia R., Petrolo E., Fucà E., Guerrera S., Valeri G., Vicari S. (2024). Stratification of Gut Microbiota Profiling Based on Autism Neuropsychological Assessments. Microorganisms.

[112] Takewaki D., Kiguchi Y., Masuoka H., Manu M.S., Raveney B.J.E., Narushima S., Kurokawa R., Ogata Y., Kimura Y., Sato N. (2024). *Tyzzerella nexilis* strains enriched in mobile genetic elements are involved in progressive multiple sclerosis. Cell Rep..

[113] Luo S., Zhao Y., Zhu S., Liu L., Cheng K., Ye B., Han Y., Fan J., Xia M. (2023). *Flavonifractor plautii* Protects Against Elevated Arterial Stiffness. Circ. Res..

[114] iMSMS Consortium (2022). Gut microbiome of multiple sclerosis patients and paired household healthy controls reveal associations with disease risk and course. Cell.

[115] Hunter S., Flaten E., Petersen C., Gervain J., Werker J.F., Trainor L.J., Finlay B.B. (2023). Babies, bugs, and brains: How the early microbiome associates with infant brain and behavior development. PLoS ONE.

[116] Feng Y., Cui Y., Jin J., Huang S., Wei J., Yao M., Zhou D., Mao S. (2023). The Alterations of Gut Microbiome and Lipid Metabolism in Patients with Spinal Muscular Atrophy. Neurol. Ther..

[117] Socała K., Doboszewska U., Szopa A., Serefko A., Włodarczyk M., Zielińska A., Poleszak E., Fichna J., Wlaź P. (2021). The role of microbiota-gut-brain axis in neuropsychiatric and neurological disorders. Pharmacol. Res..

[118] Bojović K., Ignjatović Ð.I., Soković Bajić S., Vojnović Milutinović D., Tomić M., Golić N., Tolinački M. (2020). Gut Microbiota Dysbiosis Associated with Altered Production of Short Chain Fatty Acids in Children with Neurodevelopmental Disorders. Front. Cell Infect. Microbiol..

[119] Li N., Chen H., Cheng Y., Xu F., Ruan G., Ying S., Tang W., Chen L., Chen M., Lv L. (2021). Fecal Microbiota Transplantation Relieves Gastrointestinal and Autism Symptoms by Improving the Gut Microbiota in an Open-Label Study. Front. Cell Infect. Microbiol..

[120] Bronzini M., Maglione A., Rosso R., Matta M., Masuzzo F., Rolla S., Clerico M. (2023). Feeding the gut microbiome: Impact on multiple sclerosis. Front. Immunol..

[121] Miao Z., Chen L., Zhang Y., Zhang J., Zhang H. (2024). *Bifidobacterium animalis* subsp. *lactis* Probio-M8 alleviates abnormal behavior and regulates gut microbiota in a mouse model suffering from autism. Msystems.

[122] Wang X.-Y., Meng F.-H., Zhang M.-Y., Li F.-X., Lei Y.-X., Ma Z.-G., Li J.-Q., Lou Y.-N., Chu Y.-F., Ma K. (2024). Gut *Lactococcus garvieae* promotes protective immunity to foodborne *Clostridium perfringens* infection. Microbiol. Spectr..

[123] Messaoudi S., Manai M., Kergourlay G., Prévost H., Connil N., Chobert J.M., Dousset X. (2013). *Lactobacillus salivarius*: Bacteriocin and probiotic activity. Food Microbiol..

[124] Andreozzi V., Cuoco S., Balestrieri M., Fierro F., Ferrara N., Erro R., Di Filippo M., Barbella G., Memoli M.C., Silvestri A. (2024). Synbiotic supplementation may globally improve non-motor symptoms in patients with stable Parkinson’s disease: Results from an open label single-arm study. Sci. Rep..

[125] Yang J., Li Y., Wen Z., Liu W., Meng L., Huang H. (2021). Oscillospira—A candidate for the next-generation probiotics. Gut Microbes.

[126] Gong X., Ma Y., Deng X., Li A., Li X., Kong X., Liu Y., Liu X., Guo K., Yang Y. (2024). Intestinal dysbiosis exacerbates susceptibility to the anti-NMDA receptor encephalitis-like phenotype by changing blood brain barrier permeability and immune homeostasis. Brain Behav. Immun..

[127] Thompson R.S., Gaffney M., Hopkins S., Kelley T., Gonzalez A., Bowers S.J., Vitaterna M.H., Turek F.W., Foxx C.L., Lowry C.A. (2021). Ruminiclostridium 5, *Parabacteroides distasonis*, and bile acid profile are modulated by prebiotic diet and associate with facilitated sleep/clock realignment after chronic disruption of rhythms. Brain Behav. Immun..

[128] Mohammadi F., Green M., Tolsdorf E., Greffard K., Leclercq M., Bilodeau J.F., Droit A., Foster J., Bertrand N., Rudkowska I. (2023). Industrial and Ruminant Trans-Fatty Acids-Enriched Diets Differentially Modulate the Microbiome and Fecal Metabolites in C57BL/6 Mice. Nutrients.

[129] Meng L., Jin H., Yulug B., Altay O., Li X., Hanoglu L., Cankaya S., Coskun E., Idil E., Nogaylar R. (2024). Multi-omics analysis reveals the key factors involved in the severity of the Alzheimer’s disease. Alzheimer’s Res. Ther..

[130] Langmajerová M., Ježková J., Kreisinger J., Semerád J., Titov I., Procházková P., Cajthaml T., Jiřička V., Vevera J., Roubalová R. (2025). Gut Microbiome in Impulsively Violent Female Convicts. Neuropsychobiology.

[131] Mulder D., Jakobi B., Shi Y., Mulders P., Kist J.D., Collard R.M., Vrijsen J.N., van Eijndhoven P., Tendolkar I., Bloemendaal M. (2024). Gut microbiota composition links to variation in functional domains across psychiatric disorders. Brain Behav. Immun..

[132] Zhou Y., Xu H., Xu J., Guo X., Zhao H., Chen Y., Zhou Y., Nie Y.F. (2021). *prausnitzii* and its supernatant increase SCFAs-producing bacteria to restore gut dysbiosis in TNBS-induced colitis. AMB Express.

[133] Islam T., Xu B., Bian Z. (2025). Anti-inflammatory and gut microbiota regulatory effects of ultrasonic degraded polysaccharides from *Auricularia auricula-judae* in DSS-induced colitis mice. Ultrason. Sonochem..

[134] Kim H.S., Oh S.J., Kim B.K., Kim J.E., Kim B.-H., Park Y.-K., Yang B.-G., Lee J.-E., Bae J.-W., Lee C.K. (2024). Dysbiotic signatures and diagnostic potential of gut microbial markers for inflammatory bowel disease in Korean population. Sci. Rep..

[135] Stubbendieck R.M., Hurst J.H., Kelly M.S. (2024). *Dolosigranulum pigrum*: A promising nasal probiotic candidate. PLoS Pathog..

[136] Han H.S., Soundharrajan I., Valan Arasu M., Kim D., Choi K.C. (2023). *Leuconostoc Citreum* Inhibits Adipogenesis and Lipogenesis by Inhibiting p38 MAPK/Erk 44/42 and Stimulating AMPKα Signaling Pathways. Int. J. Mol. Sci..

[137] Korona-Glowniak I., Skawinska-Bednarczyk A., Wrobel R., Pietrak J., Tkacz-Ciebiera I., Maslanko-Switala M., Krawczyk D., Bakiera A., Borek A., Malm A. (2022). *Streptococcus sobrinus* as a Predominant Oral Bacteria Related to the Occurrence of Dental Caries in Polish Children at 12 Years Old. Int. J. Env. Res. Public Health.

[138] Mochon A.B., Sussland D., Saubolle M.A. (2016). Aerobic Actinomycetes of Clinical Significance. Microbiol Spectr..

[139] Corvec S. (2018). Clinical and Biological Features of Cutibacterium (Formerly Propionibacterium) *avidum*, an Underrecognized Microorganism. Clin. Microbiol. Rev..

[140] Hou K., Wu Z.X., Chen X.Y., Wang J.Q., Zhang D., Xiao C., Zhu D., Koya J.B., Wei L., Li J. (2022). Microbiota in health and diseases. Signal Transduct. Target. Ther..

[141] Lo S.C., Hung G.C., Li B., Lei H., Li T., Nagamine K., Tsai S., Zucker M.J., Olesnicky L. (2015). Mixed group of Rhizobiales microbes in lung and blood of a patient with fatal pulmonary illness. Int. J. Clin. Exp. Pathol..

[142] Chen Y., Tang S. (2023). Gut microbiota and immune mediation: A Mendelian randomization study on granulomatosis with polyangiitis. Front. Immunol..

[143] Babar S., Liu E., Kaur S., Hussain J., Danaher P.J., Anstead G.M. (2024). *Pseudopropionibacterium propionicum* as a Cause of Empyema; A Diagnosis with Next-Generation Sequencing. Pathogens.

[144] Speirs G., Warren R.E., Rampling A. (1988). *Clostridium tertium* septicemia in patients with neutropenia. J. Infect. Dis..

[145] Pappas G., Liberopoulos E., Tsianos E., Elisaf M. (2004). *Enterococcus casseliflavus* bacteremia. Case report and literature review. J. Infect..

[146] Archambaud C., Nunez N., da Silva R.A.G., Kline K.A., Serror P. (2024). *Enterococcus faecalis*: An overlooked cell invader. Microbiol. Mol. Biol. Rev..

[147] Dunalska A., Saramak K., Szejko N. (2023). The Role of Gut Microbiome in the Pathogenesis of Multiple Sclerosis and Related Disorders. Cells.

[148] Zhou Y., Zhang L., Li Q., Wang P., Wang H., Shi H., Lu W., Zhang Y. (2024). Prenatal PFAS exposure, gut microbiota dysbiosis, and neurobehavioral development in childhood. J. Hazard. Mater..

[149] Suganya K., Koo B.S. (2020). Gut-Brain Axis: Role of Gut Microbiota on Neurological Disorders and How Probiotics/Prebiotics Beneficially Modulate Microbial and Immune Pathways to Improve Brain Functions. Int. J. Mol. Sci..

[150] Jones J., Reinke S.N., Mousavi-Derazmahalleh M., Palmer D.J., Christophersen C.T. (2022). Changes to the Gut Microbiome in Young Children Showing Early Behavioral Signs of Autism. Front. Microbiol..

[151] Chesdachai S., Yetmar Z.A., Tabaja H., Comba I.Y., Go J.R., Challener D.W., Misra A., Abu Saleh O.M. (2022). Contemporary experience of *Abiotrophia*, *Granulicatella* and *Gemella* bacteremia. J. Infect..

[152] Ryan P.M., Shin C.P. (2021). Native joint infections caused by *Parvimonas micra*. Anaerobe.

[153] Li J., Xu J., Guo X., Xu H., Huang C., Nie Y., Zhou Y. (2025). *Odoribacter splanchnicus*-A Next-Generation Probiotic Candidate. Microorganisms.

[154] Hadjiyannis Y., Ali S., Wang Q., Crawford E.C., Scholz S., Waltz P.K., Alissa F., Ayers M.H., Reyes-Múgica M., Salgado C.M. (2025). The Spectrum of Sarcina Colonization in the Gastrointestinal Tract of Pediatric and Adolescent Patients. Int. J. Surg. Pathol..

[155] Liu S., Men X., Guo Y., Cai W., Wu R., Gao R., Zhong W., Guo H., Ruan H., Chou S. (2023). Gut microbes exacerbate systemic inflammation and behavior disorders in neurologic disease CADASIL. Microbiome.

[156] Boucher M.B., Bedotto M., Couderc C., Gomez C., Reynaud-Gaubert M., Drancourt M. (2012). *Haemophilus pittmaniae* respiratory infection in a patient with siderosis: A case report. J. Med. Case Rep..

[157] Jang J.H., Yeom M.J., Ahn S., Oh J.Y., Ji S., Kim T.H., Park H.J. (2020). Acupuncture inhibits neuroinflammation and gut microbial dysbiosis in a mouse model of Parkinson’s disease. Brain Behav. Immun..

[158] Zhang X., Yu D., Wu D., Gao X., Shao F., Zhao M., Wang J., Ma J., Wang W., Qin X. (2023). Tissue-resident Lachnospiraceae family bacteria protect against colorectal carcinogenesis by promoting tumor immune surveillance. Cell Host Microbe.

[159] Chen E.Y., Mahurkar-Joshi S., Liu C., Jaffe N., Labus J.S., Dong T.S., Gupta A., Patel S., Mayer E.A., Chang L. (2024). The Association Between a Mediterranean Diet and Symptoms of Irritable Bowel Syndrome. Clin. Gastroenterol. Hepatol..

[160] Zhou X., Guo Z., Ling Y., Teng W., Cui J., Yan Z., Hou X., Cen W., Long N., Li W. (2024). Causal effect of air pollution on the risk of brain health and potential mediation by gut microbiota. Ecotoxicol. Environ. Saf..

[161] Yang Z., Liu S., Wei F., Hu J. (2024). The effects of Qingchang Ligan formula on hepatic encephalopathy in mouse model: Results from gut microbiome-metabolomics analysis. Front. Cell Infect. Microbiol..

[162] Raygoza Garay J.A., Turpin W., Lee S.H., Smith M.I., Goethel A., Griffiths A.M., Moayyedi P., Espin-Garcia O., Abreu M., Aumais G.L. (2023). Gut Microbiome Composition Is Associated with Future Onset of Crohn’s Disease in Healthy First-Degree Relatives. Gastroenterology.

[163] Kenna J.E., Chua E.G., Bakeberg M., Tay A., McGregor S., Gorecki A., Horne M., Marshall B., Mastaglia F.L., Anderton R.S. (2021). Changes in the Gut Microbiome and Predicted Functional Metabolic Effects in an Australian Parkinson’s Disease Cohort. Front. Neurosci..

[164] Bai J., Bruner D.W., Fedirko V., Beitler J.J., Zhou C., Gu J., Zhao H., Lin I.H., Chico C.E., Higgins K.A. (2020). Gut Microbiome Associated with the Psychoneurological Symptom Cluster in Patients with Head and Neck Cancers. Cancers.

[165] Fregatto L.F., Costa I.B., De Bortoli Teixeira D., Duarte J.C.M., Mascarin A.M.N., da Silveira S.B., Serva B.E.B.M., da Silva R.G., Junior F.A., Cola P.C. (2021). Oral hygiene and oral microbiota in children and young people with neurological impairment and oropharyngeal dysphagia. Sci. Rep..

[166] Wei L., Singh R., Ro S., Ghoshal U.C. (2021). Gut microbiota dysbiosis in functional gastrointestinal disorders: Underpinning the symptoms and pathophysiology. JGH Open.

[167] Chen F., Guo Z., Chen Y., Li S., Chen P. (2025). Non-alcoholic fatty liver disease enhances the beneficial effect of renal denervation on gut microbiota aberrations in rats with heart failure. BMC Microbiol..

[168] Liu Z.C., Wu D., Qu A.N., Wang L.L. (2022). Diversity and functional prediction of gut microbiota in children with autism spectrum disorder. Chin. J. Contemp. Pediatr..

[169] Krzyściak W., Jurczak A., Kościelniak D., Bystrowska B., Skalniak A. (2014). The virulence of *Streptococcus mutans* and the ability to form biofilms. Eur. J. Clin. Microbiol. Infect. Dis..

[170] Zhao Q., Baranova A., Cao H., Zhang F. (2024). Gut microbiome and major depressive disorder: Insights from two-sample Mendelian randomization. BMC Psychiatry.

[171] Kushak R.I., Winter H.S., Buie T.M., Cox S.B., Phillips C.D., Ward N.L. (2017). Analysis of the Duodenal Microbiome in Autistic Individuals: Association with Carbohydrate Digestion. J. Pediatr. Gastroenterol. Nutr..

[172] Chen Y.J., Wu H., Wu S.D., Lu N., Wang Y.T., Liu H.N., Dong L., Liu T.T., Shen X.Z. (2018). *Parasutterella*, in association with irritable bowel syndrome and intestinal chronic inflammation. J. Gastroenterol. Hepatol..

[173] Chu Y.W., Wong C.H., Chu M.Y., Cheung C.P., Cheung T.K., Tse C., Luk W.K., Lo J.Y. (2009). *Varibaculum cambriense* infections in Hong Kong, China, 2006. Emerg. Infect. Dis..

[174] Eltwisy H.O., Twisy H.O., Hafez M.H., Sayed I.M., El-Mokhtar M.A. (2022). Clinical Infections, Antibiotic Resistance, and Pathogenesis of *Staphylococcus haemolyticus*. Microorganisms.

[175] Al-Akel F.C., Chiperi L.E., Eszter V.K., Bacârea A. (2024). *Streptococcus salivarius* Role as a Probiotic in Children’s Health and Disease Prophylaxis—A Systematic Review. Life.

[176] Ojha S., Patil N., Jain M., Kole C., Kaushik P. (2023). Probiotics for Neurodegenerative Diseases: A Systemic Review. Microorganisms.

[177] Deutschmann M.W., Livingstone D., Cho J.J., Vanderkooi O.G., Brookes J.T. (2013). The significance of *Streptococcus anginosus* group in intracranial complications of pediatric rhinosinusitis. JAMA Otolaryngol. Head Neck Surg..

[178] Jangi S., Gandhi R., Cox L.M., Li N., von Glehn F., Yan R., Patel B., Mazzola M.A., Liu S., Glanz B.L. (2016). Alterations of the human gut microbiome in multiple sclerosis. Nat. Commun..

[179] Bor B., Bedree J.K., Shi W., McLean J.S., He X. (2019). Saccharibacteria (TM7) in the Human Oral Microbiome. J. Dent. Res..

